# Mycobacterial Cultures Contain Cell Size and Density Specific Sub-populations of Cells with Significant Differential Susceptibility to Antibiotics, Oxidative and Nitrite Stress

**DOI:** 10.3389/fmicb.2017.00463

**Published:** 2017-03-21

**Authors:** Srinivasan Vijay, Rashmi Ravindran Nair, Deepti Sharan, Kishor Jakkala, Nagaraja Mukkayyan, Sharmada Swaminath, Atul Pradhan, Niranjan V. Joshi, Parthasarathi Ajitkumar

**Affiliations:** ^1^Department of Microbiology and Cell Biology, Indian Institute of ScienceBangalore, India; ^2^Centre for Ecological Sciences, Indian Institute of ScienceBangalore, India

**Keywords:** mycobacteria, short cells, heterogeneity, size and density, asymmetric cell division, antibiotics, oxidative stress, nitrite stress

## Abstract

The present study shows the existence of two specific sub-populations of *Mycobacterium smegmatis* and *Mycobacterium tuberculosis* cells differing in size and density, in the **m**id-**l**og **p**hase (MLP) cultures, with significant differential susceptibility to antibiotic, oxidative, and nitrite stress. One of these sub-populations (~10% of the total population), contained **s**hort-sized **c**ells (SCs) generated through highly-deviated asymmetric cell division (ACD) of normal/long-sized mother cells and symmetric cell divisions (SCD) of short-sized mother cells. The other sub-population (~90% of the total population) contained **n**ormal/long-sized **c**ells (NCs). The SCs were acid-fast stainable and heat-susceptible, and contained high density of membrane vesicles (MVs, known to be lipid-rich) on their surface, while the NCs possessed negligible density of MVs on the surface, as revealed by scanning and transmission electron microscopy. Percoll density gradient fractionation of MLP cultures showed the **SC**s-enriched **f**raction (SCF) at lower density (probably indicating lipid-richness) and the **NC**s-enriched **f**raction (NCF) at higher density of percoll fractions. While live cell imaging showed that the SCs and the NCs could grow and divide to form colony on agarose pads, the SCF, and NCF cells could independently regenerate MLP populations in liquid and solid media, indicating their full genomic content and population regeneration potential. CFU based assays showed the SCF cells to be significantly more susceptible than NCF cells to a range of concentrations of rifampicin and isoniazid (antibiotic stress), H_2_O_2_ (oxidative stress),and acidified NaNO_2_ (nitrite stress). Live cell imaging showed significantly higher susceptibility of the SCs of SC-NC sister daughter cell pairs, formed from highly-deviated ACD of normal/long-sized mother cells, to rifampicin and H_2_O_2_, as compared to the sister daughter NCs, irrespective of their comparable growth rates. The SC-SC sister daughter cell pairs, formed from the SCDs of short-sized mother cells and having comparable growth rates, always showed comparable stress-susceptibility. These observations and the presence of *M. tuberculosis* SCs and NCs in pulmonary tuberculosis patients' sputum earlier reported by us imply a physiological role for the SCs and the NCs under the stress conditions. The plausible reasons for the higher stress susceptibility of SCs and lower stress susceptibility of NCs are discussed.

## Introduction

Bacteria maintain population heterogeneity by generating sub-populations of phenotypically different but genetically identical members with “division of labour” for the survival under diverse stress conditions (Hallez et al., 2004; Aertsen and Michiels, 2005; Zgur-Bertok, 2007; Davidson and Surette, 2008; Diard et al., 2013). Heterogeneity in cell size, culturability, morphology, and growth rate has been the hallmark of *Mycobacterium tuberculosis, Mycobacterium bovis* BCG, *Mycobacterium smegmatis*, and *Mycobacterium xenopi* cells irrespective of their habitat in *in vitro* cultures, infected macrophages, animal models, or in TB patients, under the diverse stress conditions existent in these environments (McCarthy, 1974; Nyka, 1974; Khomenko, 1987; Smeulders et al., 1999; Thanky et al., 2007; Davidson and Surette, 2008; Anuchin et al., 2009; Deb et al., 2009; Ghosh et al., 2009; Farnia et al., 2010; Ryan et al., 2010; Aldridge et al., 2012; Markova et al., 2012; Vijay et al., 2014a,b; Wu et al., 2016). The high degree of heterogeneity observed in the cell-size, morphology, growth rate, and physiology in the population of different mycobacterial species under different growth and stress conditions is suggestive of the existence of metabolically different sub-populations of cells that may have physiological relevance for survival under the respective growth and/or stress conditions.

The studies on the correlation of the differences in the physiological properties of the heterogeneous sub-populations of mycobacteria to their survival under stress conditions are beginning to emerge. High levels of lipid content was observed in the *M. tuberculosis* cells exposed to multiple stress conditions (Deb et al., 2009). Change into dormant ovoid morphology was noticed in response to severe nutrient starvation leading to gradual acidification of the culture medium (Shleeva et al., 2011). The L-shaped morphology of *M. tuberculosis* was suggested to be playing a role in the survival under stress condition (Markova et al., 2012). Differential susceptibility of sister daughter cells of *Mycobacterium smegmatis* mother cells to antibiotics could be observed due to differential growth rates (Aldridge et al., 2012). However, a later study showed that the sister daughter cells, which grew with different velocities, did not show differential antibiotic susceptibility (Santi et al., 2013). A recent live cell imaging study showed the presence of rifampicin-susceptible *M. smegmatis* cells, one of which was highly-susceptible and the other divided once but stopped further growth or division (Richardson et al., 2016).

Mild extents of cell size heterogeneity in mycobacterial populations is generated due to 70–80% of the septating *M. smegmatis, Mycobacterium marinum, Mycobacterium bovis* BCG, *M. tuberculosis*, and *Mycobacterium xenopi* cells undergoing division with 5–10% deviation of the final division site from the median generating sister daughter cells that differ 5–10% in size (Joyce et al., 2012; Santi et al., 2013; Singh et al., 2013; Vijay et al., 2014a,b), probably due to differential polar growth (Joyce et al., 2012). But a high level of cell size heterogeneity generated by the highly-deviated asymmetric cell division (ACD), with 11–31% deviation of the site of constriction from the median, produced **s**hort-sized **c**ells (SCs) and **n**ormal/long-sized **c**ells (NCs) in the ~20–30% of the septating population of *M. tuberculosis, M. smegmatis*, and *M. xenopi* cells in the mid-log phase (MLP) cultures (Vijay et al., 2014a,b). Besides the highly-deviated ACD, SCDs of short mother cells, post-elongation, would also generate SCs to contribute to the sub-population of SCs. The presence of *M. tuberculosis* SCs and NCs in the freshly diagnosed pulmonary tuberculosis patients' sputum showed the existence of the cell size heterogeneity in TB patients as well (Vijay et al., 2014a). Based on these observations, we suggested that the high levels of cell size heterogeneity in the population might have a role in stress response (Vijay et al., 2014a,b).

Following up these observations (Vijay et al., 2014a,b), in the present study, we tested whether the specific sub-populations of SCs and NCs bear differential susceptibility to antibiotic (rifampicin and isoniazid), oxidative (H_2_O_2_), and nitrite (acidified NaNO_2_) stress conditions to which both pathogenic and non-pathogenic mycobacteria are exposed both inside and outside infected cells, infected animals (Lenaerts et al., 2007; Hoff et al., 2011), TB patients (Smith, 1898; Forsyth, 1909; Summers et al., 1954; Hobby et al., 1973), and in the environment (Parashar et al., 2009; Velayati et al., 2015). For this purpose, the proportions of the SCs (formed from the highly-deviated ACD of normal/long-sized mother cells and SCDs of short-sized mother cells) and of the NCs in the MLP population of *M. smegmatis* and *M. tuberculosis* cultures, and their growth rate post-division from highly-deviated ACD of NCs and the SCD of SCs, were first determined. The SCs and the NCs were then characterized at their morphological and physiological levels. Subsequently, their stress susceptibilities against a range of concentrations of rifampicin and isoniazid (antibiotic stress), H_2_O_2_ (oxidative stress), and acidified NaNO_2_ (nitrite stress) were determined. All the data put together showed that the two specific sub-populations of *Msm* and *Mtb* cells, the SCs and the NCs, which differed not only in cell size but in cell density also, showed consistent, reproducible and significant differential susceptibility, irrespective of their comparable growth rates, to all the four different types of stress agents having different targets and modes of action.

## Materials and methods

### Bacterial strains and growth conditions

*M. smegmatis* mc^2^155 (*Msm*) (Snapper et al., 1990) and *M. tuberculosis* H_37_R_a_ (*Mtb*) (Supplementary Table S1) were grown in Middlebrook 7H9 broth without or with ADS (albumin, dextrose, sodium chloride) supplement, respectively, and with 0.05% Tween 80, at 37°C, with shaking at 170 rpm, till the OD_600nm_ of the culture reached 0.6 (mid-log phase; MLP). *Msm* cells were also grown in Youmans and Karlson's medium supplemented with 0.2% v/v Tween 80 (Youmans and Karlson, 1947), Dubos broth base (Difco) (Dubos et al., 1950), and Sauton's medium (Himedia) (Sauton, 1912) under the same conditions. Mycobacterial cells were plated on Middlebrook 7H10 agar and incubated at 37°C to determine cfu. The *Msm* colonies were counted after 3–4 days of incubation at 37°C. In the case of *Mtb*, the colonies were counted after about 25 days of incubation in carbon dioxide incubator at 5% CO_2_, 95% humidity, and 37°C.

### Determination of the proportion of different size cells in mycobacterial mid-log phase (MLP) population

*Msm* and *Mtb* cells (1 ml each) from the respective MLP culture were pelleted down at ~3,900 × g for 10 min at 25°C, washed with 1 ml of 1x PBS, and centrifuged at ~3,900 × g for 10 min at 25°C. The cell pellet was then resuspended in 200 μl of 4% paraformaldehyde (Vijay et al., 2014a,b) and incubated for 1 h at 25°C following which the cells were pelleted down at ~3,900 × g for 10 min at 25°C. The pellet was finally resuspended in 400 μl of 1x PBS. The cells from 20 μl of this cell suspension was then adhered to poly-L-lysine coated multi-well slides, washed once with 1x PBS for 1 min at 25°C in the multi-well slide itself, and mounted in 90% glycerol. The images were captured under DIC using Carl Zeiss AXIO Imager M1 microscope and the sizes of a large number of *Msm* (*n* = 343) and *Mtb* cells (*n* = 389) were measured using AxioVision 4 software following which the proportions of differently-sized cells in MLP were calculated. The proportions of the cells differing in size at an increment of 1 μm were plotted against the size ranges.

### Determination of the proportion of *Msm* short cells generated by different types of cell division in MLP population

**Figure 1:**
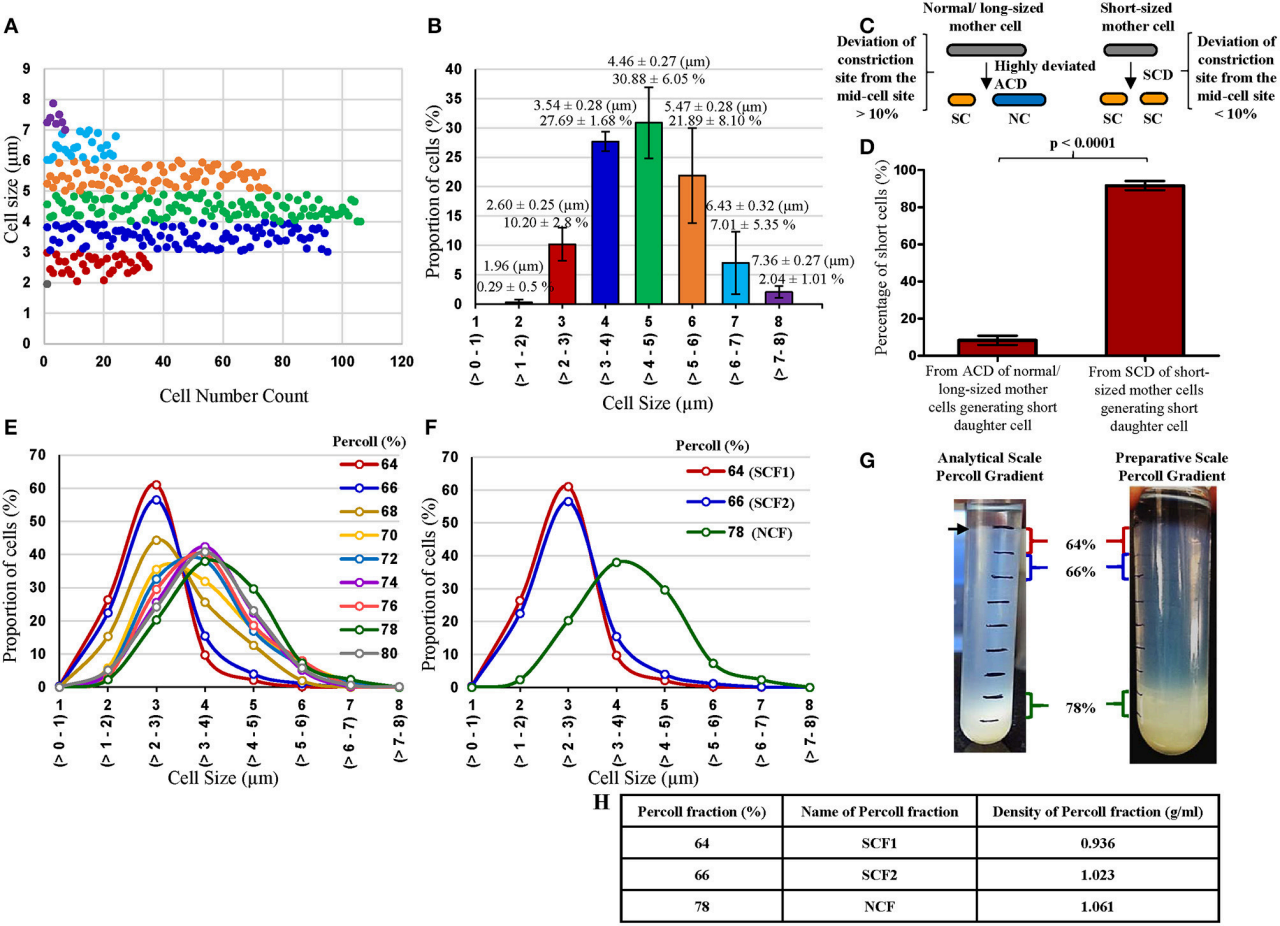
**Profile of cell-size-based subpopulations of *Msm* from MLP, proportion of short cells in the MLP generated from different types of cell division and Percoll gradient fractions**. **(A)** Different subpopulations of cells from *Msm* mid-log phase with specific range of cell lengths. Each dot represents one cell (*n* = 343). **(B)** Quantitation of the proportion of the cells in each subpopulation shown in **(A)** (*n* = 3). **(C)** Diagrammatic representation of the short cells (SCs) generating from highly deviated asymmetric division of normal/long-sized mother cells (NC) and from symmetric cell division (SCD) of short sized mother cells. **(D)** Quantitative representation of the proportion of SCs generated from the 2 types of cell divisions: asymmetric cell division (ACD) of NC mother cells and SCD of short mother cells (*n* = 1,066 cells from 533 divisions). The length of the SC was taken as ≤ 2.6 μm, as measured from the MLP under DIC shown in **(A,B)**. **(E)** Length distribution of *Msm* cells from all the Percoll fractions (*n* ≥ 300). **(F)** Length distribution of *Msm* cells from 64, 66, and 78% Percoll fractions (*n* ≥ 300). The values in brackets on the X-axis show the size range of the cells in each group. **(G)** Profile of *Msm* analytical and preparative scale Percoll gradient after centrifugation, showing a thick band at 78% Percoll fraction. The arrow indicates the thin band of SCs in the 64% of the analytical scale Percoll gradient. **(H)** Densities of the Percoll fractions enriched in SCs (64 and 66%) and NCs (78%).

The *Msm* MLP cells were pelleted down and fixed using 4% paraformaldehyde, as mentioned above. DIC images were taken using Carl Zeiss AXIO Imager M1 microscope and the sizes of a large number of *Msm* cells undergoing division (*n* = 533) were measured using AxioVision 4 software. The average length of short cells (2.60 μm) in the MLP population, as presented in Figures 1A,B, was kept as the parameter to identify the short cells for determining the proportion of short cells generated through different types of cell division. These are: (i) highly-deviated ACD of normal/long-sized mother cells and (ii) symmetric cell division (SCD) of short mother cells.

### Percoll gradient centrifugation for the size fractionation of *Msm* and *Mtb* cells

For the size fractionation of *Msm* and *Mtb* cells, discontinuous gradients from 64 to 80% and 60 to 76%, respectively, at 2% increment of the percentage of Percoll, were set up. Stock Isotonic Percoll (SIP), was prepared by mixing nine parts of Percoll (Sigma) with one part of sterile 1.5 M NaCl. In order to obtain the different percent Percoll fractions (64–80% for *Msm* and 60–76% for *Mtb* cells), the SIP was further diluted by adding 0.15 M NaCl to obtain a final volume of 1 ml for each fraction. One ml each of the above mentioned respective percentage of SIP was pipetted into ultracentrifuge tube (13.2 ml tube volume; Beckman Coulter, Cat. No. 344059).

*Msm* or *Mtb* cells from 50 or 100 ml culture, were harvested at 0.6 OD_600nm_ by centrifugation at ~4,300 × g, 25°C for 15 min. The cell pellet was washed twice with 5 ml of sterile 0.5% Tween 80 and centrifuged at ~4,300 × g, 25°C for 15 min. The cell pellet was resuspended in 1 ml of 0.5% sterile Tween 80, vortexed for 1 min, syringed (one ml tuberculin syringe) to remove clumps, if any, and was layered onto the top of the gradient. The cells were fractionated into SCF and NCF by centrifugation in SW41 rotor in Beckman L8-70M ultracentrifuge at 385 × g for 1 h at 20°C. Following the centrifugation, the cells from each percentage fraction of Percoll were pipetted out and diluted 5 times with 1x PBS. The samples were then pelleted down at ~3,900 × g, 25°C for 10 min.

The cells were acid fast stained and examined under bright-field and DIC using Carl Zeiss AXIO Imager M1 microscope, to ensure purity of the preparation and to verify the size distribution of cells. The purity checking and verification of size distribution of Percoll fractions were carried out every time for every fresh fractionation of the cells. The preparative scale Percoll gradient fractionation was also carried out in the same way as that for analytical scale fractionation except that the volume of the culture used for harvesting was 200 ml and the total volume of Percoll fractions used was 36 ml in a 50 ml polypropylene screw-capped tube (Beckman Coulter, Cat No. 357002) in Beckman JS-13.1 rotor in J30-I model Beckman high speed centrifuge at ~350 × g, 20°C for 1 h.

### Determination of the regeneration potential, heat sensitivity, and viability of SCs and NCs

The cells from SCF1 and SCF2 mixture and from NCF were independently inoculated into fresh Middlebrook 7H9 medium as well as plated on Middlebrook 7H10 agar medium, and examined the cells from MLP culture and from colonies from the plates, under microscope (DIC). Heat susceptibility was verified at 75°C for 15 min, followed by plating on Middlebrook 7H10 agar, with an equivalent number of heat-unexposed control cells, as mentioned (Traag et al., 2010) and cfu was determined. Vital staining was carried out using commercially available viable/nonviable cell staining kit (LIVE/DEAD® *Bac*Light™, Molecular Probes, Invitrogen, USA), containing a combination of SYTO9 and propidium iodide (Stocks, 2004; Lahiri et al., 2005). The stained cells were viewed under Carl Zeiss AXIO Imager M1 microscope and counted for SYTO9-positive and PI-positive cells (*n* = >300 cells each).

### Sample preparation for scanning electron microscopy (SEM)

The cells from *Msm* SCF1 and SCF2 mixture and from NCF were washed once with 1 ml of 1x PBS and centrifuged at ~3,900 × g for 10 min at 4°C, fixed with 2% glutaraldehyde, treated with 0.5% osmium tetroxide for 2 h, dehydrated in graded series of ethanol 30, 50, 70, and 100%. The samples were then sputter-coated with gold and observed under SIRION scanning electron microscope and the images were captured, as described (Vijay et al., 2014b).

### Sample preparation for transmission electron microscopy (TEM)

The cells from *Msm* SCF1 and SCF2 mixture and from NCF were taken and processed for transmission electron microscopy (TEM), as described (Takade et al., 1983), with minor modifications (Vijay et al., 2012). The cell pellet was suspended in 2% (v/v) glutaraldehyde (Sigma) for a period of 20 min before pre-fixation with osmium tetroxide (Sigma). The cells were washed with the same buffer and pre-fixed with 1% (w/v) osmium tetroxide buffered with 0.15 M cacodylate buffer for 1 h at 25°C. The pre-fixed cells were then washed with the same buffer and post-fixed for 2 h at 25°C in 0.15 M sodium cacodylate (Sigma) buffer containing 2% (w/v) tannic acid (Sigma) and 2% (v/v) glutaraldehyde. After washing with the same buffer, the cells were re-fixed in 1% (w/v) osmium tetroxide overnight at 4°C. The cells were then dehydrated in graded series of 20, 30, 50, 70, and 90% ethanol solutions (prepared from 95% ethanol; Merck), and then infiltrated with 70% LR white resin overnight. Following infiltration, embedding was performed with 100% LR white resin and subsequently, curing was effected at 62°C for 48 h. The blocks were trimmed using ultramicrotome and ultra-thin sections of 70 nm thickness were cut and stained with 0.5% uranyl acetate, 0.4% lead citrate and observed under transmission electron microscope at 120 kV (BioTwin, FEI).

### Exposure of the mycobacterial cells to antibiotic, oxidative, and nitrite stress

The *Msm* cells from the different Percoll fractions (64, 66, and 78%) were suspended in 400 μl of 1x PBS or 0.5% Tween 80 or Middlebrook 7H9 medium (did not make a difference in the results) and exposed to different stress agents. In order to obtain 10^3^ cells/ml of the analytical scale Percoll gradient fractionated *Msm* SCF1, SCF2, and NCF cells (NCF was further diluted 250 times) (64, 66, and 78% Percoll fractions, respectively) for exposure to stress, 200 μl from each of the respective cell suspensions was added into 25 ml Middlebrook 7H9 medium taken in 100 ml flask followed by exposure to stress agents as mentioned below.

In order to obtain 10^5^ cells/ml of the preparative scale Percoll gradient fractionated SCF1 and SCF2 samples, the cells from the 64 and 66%, respectively, were initially pooled in 400 μl of 1x PBS or 0.5% Tween 80 or Middlebrook 7H9 medium. Further, the samples from the 78% Percoll fraction (NCF), following resuspension in 400 μl of 1x PBS or 0.5% Tween 80 or Middlebrook 7H9 broth, were further diluted with Middlebrook 7H9 broth to visually obtain the same cell density as that of pooled SCF cells (see Supplementary Text). Subsequently, 100 μl from each of the respective cell suspensions was added into 25 ml Middlebrook 7H9 medium taken in 100 ml flask followed by exposure to stress agents as mentioned below.

#### Antibiotic stress

The response to antibiotics was determined by exposing the fractions (10^3^ cells/ml) to different concentrations of rifampicin (25, 50, 75, 100 μg/ml) for 4 h or of isoniazid (2.5, 5, 7.5, 10, 15 μg/ml) for 6 h. Subsequent to the determination of the rifampicin or isoniazid concentration which results in 50% survival of the cells, the Percoll fractions were then exposed independently to the same concentration for different durations (2, 4, and 6 h) for rifampicin and (3, 6, and 9 h) for isoniazid separately.

#### Oxidative stress

The oxidative stress was applied using a range (0.4–1 mM) of H_2_O_2_ concentrations on 10^3^ or 10^5^ *Msm* cells per ml of SCF1, SCF2 and NCF for 30 min or 1 h, in shaking cultures in Middlebrook 7H9 broth at 170 rpm and 37°C, as described (Milano et al., 2001). As H_2_O_2_ is known to decompose upon storage, the concentration of the stock solution of H_2_O_2_ was routinely determined by checking the absorbance at 240 nm (Beers and Sizer, 1952) before using it for experiments. The durations of the exposures of the cells to H_2_O_2_ were well within the 80 min where the decomposition rate of H_2_O_2_ was undetectable under sterile conditions (Spain et al., 1989). The concentration of H_2_O_2_ during the course of oxidative stress was estimated using the xylenol orange assay.

In the case of *Mtb* cells, the SCF 1 (60+62% Percoll fraction), SCF2 (64% Percoll fraction) and NCF (66% Percoll fraction) were initially visually made to same cell density and 100 μl of each of the cell suspension was added into 25 ml Middlebrook 7H9 medium taken in 100 ml flask. Subsequently, the cfu was determined by plating. After such multiple trials, it was found that each ml in the 25 ml 7H9 medium contained ~10^4^ cells. In this manner, 10^4^ cells/ml of these Percoll fractions were exposed to 0.4, 0.8, and 1.2 mM H_2_O_2_ for 24 h, in Middlebrook 7H9 broth with ADS supplement (catalase was not used in the supplement) in shaking cultures at 170 rpm and 37°C.

#### Nitrite stress

In the case of nitrite stress, the cell samples (10^3^ cells/ml) were exposed to 7.5 mM of acidified (pH 5) sodium nitrite for 30 min, in shaking cultures at 170 rpm and 37°C, as described (Colangeli et al., 2009) (Supplementary Text). This concentration of acidified nitrite was chosen as it gave about 50% lethality such that the percentage of survival of both NCs and SCs could be determined. This concentration was selected after checking the percentage of survival at various other (2.5, 3.75, 5, 6.25, 8.75, and 10 mM) concentrations lower and higher than 7.5 mM.

The percentage survival against each kind of stress agent was determined by plating the cells on stress-free Middlebrook 7H10 agar, under different dilutions, at 0 h (before exposure) and at the time mentioned post-exposure to the respective stress agent.

### H_2_O_2_ assay using xylenol orange

Prior to use, concentration of H_2_O_2_ was routinely estimated using the (ferrous ammonium sulfate/xylenol orange) FOX reagent (Rhee et al., 2010). The FOX reagent was prepared by mixing reagent A (25 mM ferrous ammonium sulfate in 2.5 M H_2_SO_4_) and reagent B (100 μM xylenol orange in 100 mM sorbitol) in a 1:100 ratio (v/v), respectively, based on Pierce quantitative peroxide assay kit. Fifty microliter of the sample containing H_2_O_2_ was added into 950 μl of FOX reagent and incubated at 25°C, in the dark, for 30 min. The absorbance was then taken at 560 nm and the concentration of H_2_O_2_ in the samples was calculated from a standard curve generated using known concentrations of H_2_O_2_.

### Live cell time-lapse microscopy of unstressed and stressed cells

Live cell time-lapse microscopy of *Msm* cells was performed using modification of the agarose pad method, as described (Vijay et al., 2014a). In brief, Middlebrook 7H9 medium containing 1.9% low melting point agarose (Sigma-Aldrich) was used to form an agarose pad on a glass slide. After solidification, a portion of the agarose (about 1/5th of the total agarose pad area) was cut out using a blade to make a well for the introduction and removal of the stress agent. Ten microliter of mid-log phase *Msm* cells were placed on top of the agarose pad, covered by cover slip and kept at 37°C for 1 h. This slide was observed under Carl Zeiss AXIO Imager M1 microscope using live cell imaging option, with Z-stacking at 37°C. Individual cells were observed and the DIC images were taken at every 10 min time interval. Immediately after the first division, 12.5 μg/ml rifampicin or 0.8 mM H_2_O_2_ was added, replaced with the medium after 50 or 60 min, respectively and observed further until the next division. The data was analyzed and video tracking was made using AxioVision 4 software and ImageJ version 1.43 m.

### Statistical analysis of the survival against antibiotic, oxidative, and nitrite stress

Both parametric and non-parametric tests were used for evaluating the statistical significance of the pairwise differences in the survival rates and the results were comparable from both the analyses. The values obtained from parametric tests have been mentioned in the figures since nonparametric tests have lower power (Motulsky, 2007). When the computed survival rates exceeded 100%, the values were rounded off to 100%. When data from 10 different experiments was available, the two-tailed paired *t*-test, using GraphPad Prism software, was employed, as the survival of NCF cells needed to be compared with that of SCF1 or SCF2 cells obtained from the same culture. If the survival rate of one of the pairs was greater than the other in all the 10 or at least 9 of the 10 experiments (biological replicates), it was considered to be statistically significant by the binomial test.

## Results

### Experimental basis, rationale and strategy

In order to study the natural cell size heterogeneity involving the SCs and the NCs in *Msm* and *Mtb* MLP cultures and their role in stress response, specific sub-populations of cells that differed in cell length were first identified in the cultures. The length of the cells was measured from the DIC microscopy images of *Msm* and *Mtb* cells in the MLP cultures to get a profile of the natural distribution of the cell lengths in the cultures. The cells were then categorized into sub-populations in terms of cell length differences by plotting the proportion of cells against cell lengths over a range of <1 μm upto >8 μm with the cell lengths differing by an increment of 1 μm. Then, the proportions of SCs and NCs in the population, which might have been formed by the SCDs of NCs, highly-deviated ACDs of NCs, where the lengths of the sister daughter cells differed by 1 μm or more, and by the SCDs of SCs, were determined. Subsequently, the viability of SCs and NCs and their independent potential to regenerate an entire MLP population were determined to ensure that they were viable and growing and that they were not dormant cells, spores or VBNCs.

Further to these characterisations, in order to determine the individual susceptibility/tolerance of the SCs and the NCs to stress conditions, the MLP cultures were fractionated using Percoll gradient centrifugation, to get **SC**-enriched **f**ractions (SCF) and **NC**-enriched **f**ractions (NCF). The SCF and the NCF cells were then exposed individually for different durations to a range of concentrations of rifampicin and isoniazid (antibiotic stress), H_2_O_2_ (oxidative stress), and acidified sodium nitrite (nitrite stress), which are the stress agents having different targets and modes of action that are usually faced by mycobacteria. The cfu of the samples were determined before and after the exposure to find out their individual susceptibility/tolerance to the stress conditions. In parallel, using live cell imaging, the susceptibility/tolerance of the individual members of the following three different sister daughter pairs of cells was determined in terms of the time taken by the individual sister daughter cells for the growth and next division post-exposure to the stress: (i). the SC-NC pairs of sister daughter cells, formed from the highly-deviated ACDs of normal/long-sized mother cells; (ii). the SC-SC pairs of sister daughter cells formed from the SCDs of short-sized mother cells; and (iii). the NC-NC pairs of sister daughter cells formed from the SCD of normal/long-sized mother cells. Each experiment was repeated atleast 10 times with atleast 10 different individual independent preparations of the samples from the MLP cultures of *Msm* and *Mtb* cells. The statistical significance of the data were analyzed.

### Existence of cell-size based sub-populations in *Msm* MLP cultures

Examination of the Middlebrook 7H9 medium grown *Msm* MLP cells (*n* = 343 cells) using DIC microscopy showed the presence of size-wise distinct subpopulations of cells (Figures 1A,B). The cells were grouped into short and normal-sized cells in an unbiased manner purely based on the clear segregation of the cells in terms of their size observed in the population depicted in Figures 1A,B. The clear demarcation of the subpopulation of cells (~10%) in the size range of 2–3 μm (2.60 ± 0.25 μm) was called the **s**hort-sized **c**ells (SCs) (Figures 1A,B). Likewise, the distinct major proportion (~80%) of the cells in the 3–6 μm size range (4.46 ± 0.27 μm) was termed the **n**ormal-sized **c**ells (NCs) (Figures 1A,B). These lengths of SCs and NCs corresponded to the lengths of the SCs and NCs formed as sister daughter cells immediately post-constriction during cell division and to the sizes of short and long portions, respectively, of the asymmetrically constricted *Msm* cells documented earlier (Vijay et al., 2014b).

Thus, the MLP population clearly contained atleast two distinct sub-populations of cells of different size, the SCs and the NCs. There were also very low proportions of very short cells and very long cells at either end of the cell-size spectrum, revealing the high levels of cell-size heterogeneity in *Msm* MLP population. The proportions of the SCs and the NCs in the *Msm* MLP populations grown in Youmans and Karlson's medium (synthetic medium; Youmans and Karlson, 1947), Dubos broth base (Dubos et al., 1950), and Sauton's medium (Sauton, 1912) were also about 12–16% and 80–90%, respectively, of the total population (Supplementary Table S2), like in the case of the MLP population in the Middlebrook 7H9 medium.

Based on the data from Figures 1A,B on the length criterion of SCs and NCs, DIC microscopy of 4% paraformaldehyde fixed division-constriction completing cells (*n* = 533 divisions, generating 1,066 sister daughter cells) showed that 8.34 ± 2.45% of the SCs came from the highly-deviated ACD of NCs [with the division constriction site deviating more than 10% (11–31%) from the median], as reported earlier by us (Vijay et al., 2014b) (Figures 1C,D). The remaining 90–92% of the SCs came from the SCD of short mother cells generating two SCs with minor size differences (with the division constriction site deviating <10% from the median) (Figures 1C,D). Thus, SCD of short mother cells is the largest contributor of SCs, with the highly-deviated ACD of NCs being the least contributor, to the total population of *Msm* SCs in the culture.

### Enrichment of SCs and NCs for exposure to stress

Consistent with the distinct proportions of SCs and NCs obtained from the *Msm* MLP population (Figure 1B), Percoll gradient fractionation of *Msm* MLP cells and measurement of cell length in different Percoll fractions yielded essentially a bimodal distribution (Figures 1E,F; Supplementary Tables S3, S4; Supplementary Text). While the *Msm* SCs partitioned into the lower buoyant density Percoll fractions (SCF1 & SCF2; enriched in SCs), the *Msm* NCs went into the higher buoyant density Percoll fractions (NCF; enriched in NCs) (Figures 1G,H; Supplementary Tables S3, S4; Supplementary Text). The average lengths of the *Msm* SCF cells in the low density Percoll fractions [64% (SCF1) & 66% (SCF2)] were comparable to the lengths of the SCs (2–3 μm; 2.60 ± 0.25 μm) from the earlier measurements of MLP cells (Figures 1A,B,E,F, respectively; Supplementary Tables S3, S4). Similarly, the majority of *Msm* NCF cells (~70%) in the high density Percoll fractions (76–80%) showed an average length comparable to that of the NCs (3–6 μm; 4.46 ± 0.27 μm) from the earlier measurements of MLP cells (Figures 1A,B,E,F, respectively; Supplementary Tables S3, S4). The slightly lesser size of the cells given in Supplementary Tables S3, S4 (from Percoll fractions), as compared to those in Figures 1A,B (from MLP cells before Percoll fractionation), might be due to shrinkage during acid-fast staining prior to the length measurements of the cells in the Percoll fractions. The preparative scale fractionation of *Msm* cells also showed similar distribution of SCF1, SCF2, and NCF cells (Supplementary Table S5).

The Percoll fractions between the SCF2 (66%), NCF (78%), and 80% contained cells of sizes steadily increasing from that of SCs to that of NCs, without enrichment of cells of any particular size, and hence called *Msm* **m**ixed-sized **c**ells' **f**raction (MCF). The cells in *Msm* SCF1, SCF2, and NCF also contained outliers in cell length. Besides the major proportion of SCs, SCF1 and SCF2 also contained low proportions of cells of lengths much higher (>3 μm) than those in SCF1 and SCF2. Similarly, though the NCF mostly contained NCs, small proportion of cells of lengths much lower (<3 μm) than those in NCF were also present (Figures 1E,F; Supplementary Tables S3, S4). Thus, there were low density outlier longer cells in SCF1 and SCF2, and high density outlier short cells in the NCF. It was possible that the low buoyant density long-sized cells in the SCF1 and SCF2 were the shorter-sized daughter cells formed from the highly-deviated ACD of long mother cells, as we have observed mother cells of length ≥9 μm. Similarly, the high buoyant density short-sized cells in the NCF might be the normal-sized cells formed from the highly-deviated ACD of short-sized mother cells. These outliers, having partitioned into the same Percoll fractions as SCF or NCF cells, respectively, have the same buoyant density as that of SCF and NCF cells. These cells were also included in the determination of the average length of the cells in the respective Percoll fraction. Thus, the low density of SCs was not due to low weight expected to be associated with short size. Similarly, the high density of NCs was not due to higher weight expected to be associated with larger size. These observations indicated that molecular level differences exist between the SCs and the NCs. It was not possible to distinguish between the major proportion of SCs fractionating into the low density SCF1 and SCF2 and the minor proportion of SCs partitioning into the high density NCF in terms of the two modes of their generation (ACD of NCs and SCD of SCs; see Figure 1C) in the population. Conversely, it was not possible to distinguish between the major proportion of NCs fractionating into high density NCF and the minor proportion of NCs fractionating into low density SCFs, in terms of whether the NCs have come from the highly-deviated ACD of normal/long-sized mother cells or from the SCD of normal/long-sized mother cells.

### Morphological and physiological characterisation of SCs and NCs

**Figure 2:**
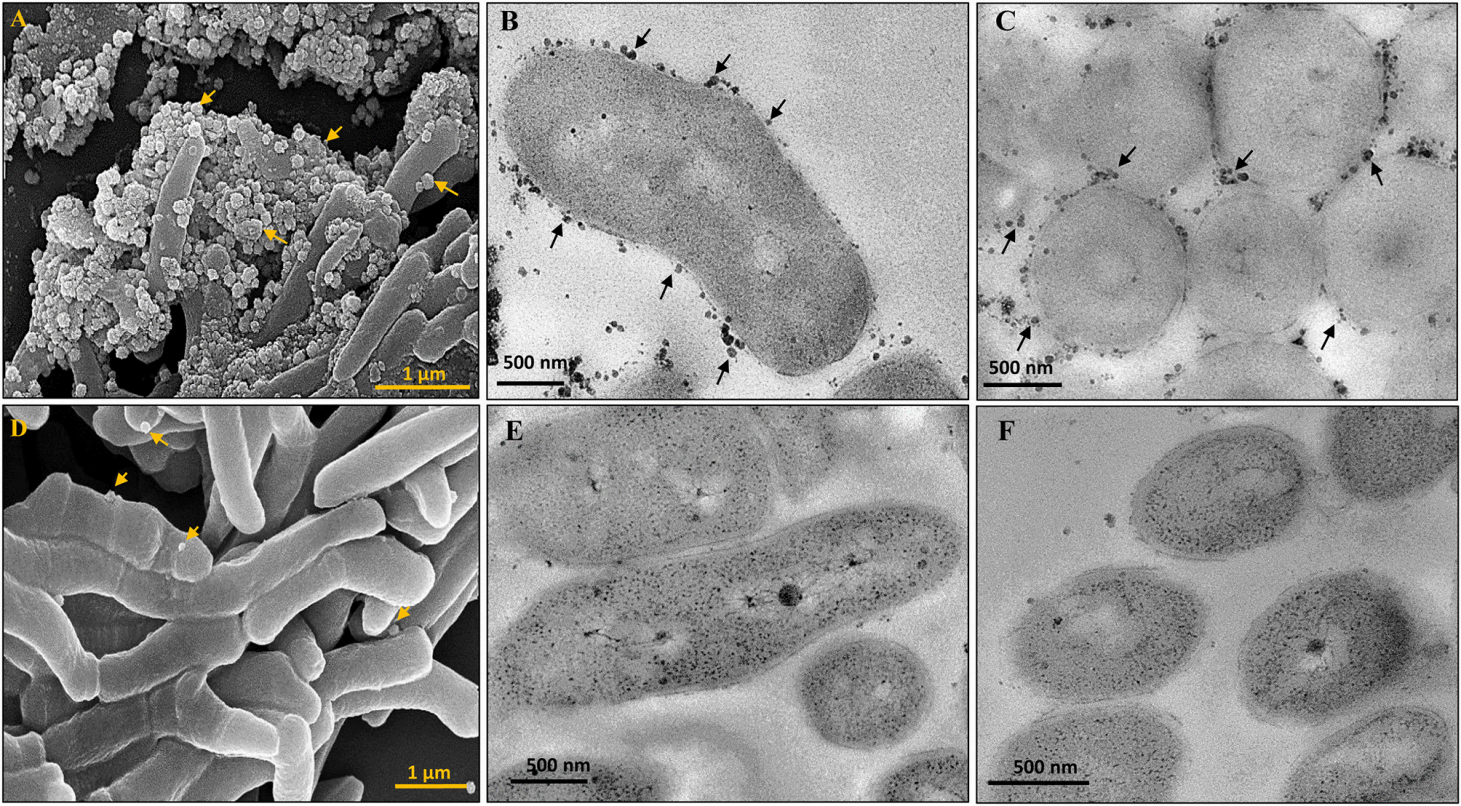
**Scanning and Transmission electron micrographs of cells in *Msm* SCF and NCF**. MVs on SCF cells visualized by **(A)** SEM, **(B,C)** Longitudinal and transverse sections by TEM, respectively. NCF cells visualized by **(D)** SEM, **(E,F)** Longitudinal and transverse sections by TEM, respectively. Arrow head indicates MVs.

The presence of lipid-rich membrane vesicles (MVs) on mycobacterial cells from a whole population, but not sub-populations, have earlier been reported (Prados-Rosales et al., 2011). Interestingly, morphological characterisation of *M. smegmatis* SCF and NCF cells using scanning and transmission electron microscopy showed the presence of high density of MVs on the surface of SCF cells as compared to the negligible density of the MVs on the surface of NCF cells (Figures 2A–F).

**Figure 3:**
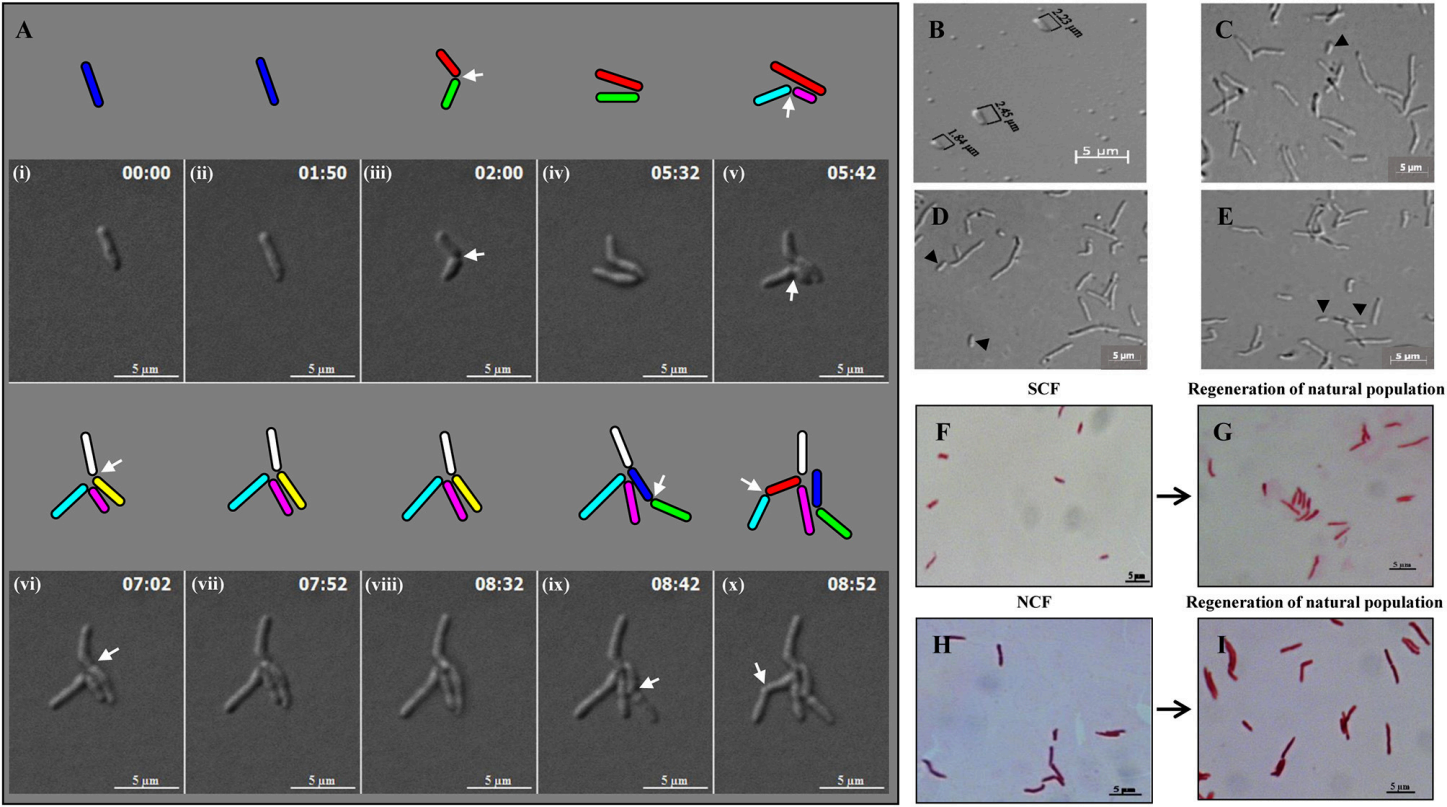
**Panels from Video S1 on the live cell imaging of *Msm* short cells generating micro colony and population regeneration potential of the cells in the SCF and NCF**. **(A)** An *Msm* cell elongates and divides symmetrically to generate two short daughter cells. One of the short daughter cells (green), grew and divided asymmetrically to generate two unequal-sized daughter cells (indicated in cyan and pink). The other short daughter cell (red), grew and divided symmetrically to generate two comparably-sized short daughter cells (indicated in white and yellow). The daughter cells from the earlier asymmetric division (cyan) further divided symmetrically to generate two daughter cells. The short daughter cell (yellow) further grew and divided symmetrically to generate two daughter cells. Arrows show the site of constriction. Cells of length ≤ 2.60 ± 0.25 μm were considered short cells (as per Figure 1B). **(B)** Fraction enriched for *Msm* short cells. **(C)** Cells from mid-log phase population. **(D)** Population generated from the short-cells-enriched fraction after reinoculation into Middlebrook 7H9 liquid medium. **(E)** Population generated from plating of the cells in the short-cells-enriched fraction on Middlebrook 7H10 agar. The compositions of the cells in **(D,E)** are similar to that in **(C)**. Arrows indicate short cells. Length of *Msm* short cells are shown in **(B)**. **(F–I)** Acid-fast stained cells in the SCF and NCF and the respective population generated from them after reinoculation into Middlebrook 7H9 media and incubated till the cultures reached 0.6 OD_600nm_.

Live cell imaging of *Msm* MLP cells showed growth (elongation) followed by SCD and ACD of SCs and their daughter cells to generate microcolony (Video S1; Figure 3A). The cells from SCF1 (Figures 3B,F), upon reinoculation into fresh Middlebrook 7H9 medium and upon directly plating on Middlebrook 7H10 agar, gave rise to populations (Figures 3D,E, respectively), which were similar in composition to the MLP population (Figures 3C,G,I), containing about 10% SCs and 90% NCs (*n* = 300). The NCF cells also could regenerate MLP-like population upon reinoculation into fresh medium (Figures 3H,I).

The analysis of live cell imaging data for the growth and division lineages and durations of SCs and NCs in the MLP population showed that the average time taken by these cells post-birth for growth and next division were 3.64 and 3.66 h, respectively (*n* = 10). Live cell imaging of the growth and division, and not the flask-based culture, was the only method by which the growth rates of SCs and NCs could be determined on an individual cell basis as the SCs and the NCs would otherwise give rise to a normal MLP-like population when they are grown in flask. About 99% of the cells in the SCF1 and SCF2 were found viable, upon SYTO9/propidium iodide (SYTO9/PI) vital staining (Stocks, 2004; Lahiri et al., 2005) (data not shown). The SCF and the NCF cells were acid fast positive (Figure 3F) and lost viability upon exposure to 75°C for 15 min (Traag et al., 2010), as verified by plating of the cells, post-exposure to heat, on Middlebrook 7H10 agar.

### Significant differential susceptibility of SCF and NCF cells to antibiotic, oxidative, and nitrite stress

**Figure 4:**
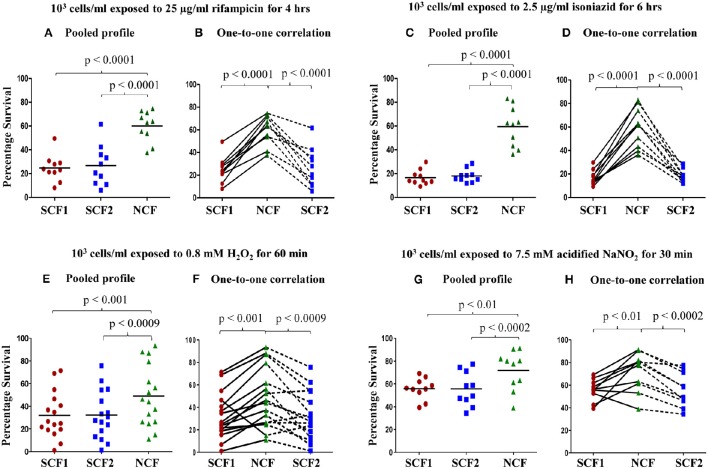
**Percentage survival of *Msm* SCF1, SCF2, and NCF cells, when plated after exposure to different stress conditions**. Percentage survival of 10^3^ cells/ml of SCF1 and SCF2 in comparison to NCF when exposed to: **(A,B)** 25 μg/ml rifampicin for 4 h; **(C,D)** 2.5 μg/ml isoniazid for 6 h; **(E,F)** 0.8 mM H_2_O_2_ for 60 min; **(G,H)** 7.5 mM NaNO_2_(pH 5) for 30 min. **(A,C,E,G)** Pooled results of all the experimental sets (*n* ≥ 10 samples each); **(B,D,F,H)** Relative one-to-one correlation of the survival of the samples in each independent set.

Based on the indication that there might exist physiological differences between the SCF and NCF cells, their susceptibility to antibiotic, oxidative, and nitrite stress were determined. The SCF1 and SCF2 cells showed significantly low percentage survival than an equivalent number of NCF cells when exposed to rifampicin (25 μg/ml for 4 h), isoniazid (2.5 μg/ml for 6 h), H_2_O_2_ (0.8 mM for 60 min), and acidified sodium nitrite (7.5 mM for 30 min) at 10^3^ cells per ml density. The pooled profiles of atleast *n* = 10 sets of samples against each stress agent, where each set consisted of SCF1, SCF2, and NCF cells that were Percoll fractionated from an independent fresh MLP culture, are shown (Figures 4A,C,E,G, respectively). Identical behavior was observed for the SCF1, SCF2, and NCF cells against a range of concentrations of rifampicin (50, 75, and 100 μg/ml), isoniazid (5.0, 7.5, 10, and 15 μg/ ml), and H_2_O_2_ (0.5–0.9 mM), irrespective of different periods of exposure and different densities of the cells (Supplementary Figures S1–S3, respectively).

Interestingly, the profiles of one-to-one correlation of the survival of SCF1, SCF2, and NCF cells of each sample set (SCF1, SCF2, and NCF cells from each independent culture) showed that the extent of survival against each of the stress agent was found to vary for each of the fractions, irrespective of the concentrations of the stress agent or period of exposure or cell density (Figures 4B,D,F,H, respectively). As a representative example for such variations in the response of the cells to rifampicin, the extent of difference in the survival of individual SCF1, SCF2, and NCF cells and of NCF cells as compared to the SCF1 and SCF2 cells, showed variations among the 10 sample sets (Figures 4A,B and Supplementary Figures S1A–F). Similarly, as a representative example for such variations in the response of the cells to H_2_O_2_, in all the experiments of a total of 80 sets (*n* = 80 sets) performed against oxidative stress using different concentrations of H_2_O_2_, different cell densities and different periods of exposure (Figures 4E,F and Supplementary Figures S3A–N), the extent of survival of SCF1, SCF2, and NCF cells has always shown variations. These observations indicated that probably the processes involved in the survival of the bacilli against antibiotic, oxidative and nitrite stress may be stochastic in nature (see Discussion). Strangely, in about 1–10% of such sets of samples, SCF1 and SCF2 cells showed higher survival than the NCF cells from the same culture, against H_2_O_2_ (out of *n* = 80 sets total; Figure 4F and Supplementary Figures S3B,D,H), acidified nitrite (out of *n* = 10 sets total; Figure 4H), rifampicin (out of *n* = 70 sets total; Supplementary Figure S1H), and isoniazid (out of *n* = 80 sets total; Supplementary Figure S2J). At present, we do not have an explanation for such contradictory response.

In spite of the variations in the extent of survival of SCF1, SCF2, and NCF cells against the different stress agents, the technical triplicates of each set of samples involving SCF1, SCF2, and NCF cells were consistently comparable and the nature and the trend of the response of the individual fractions to all the four stress agents were reproducible, consistent and statistically significant at different cell densities and different durations of exposure. At all the selected range of concentrations of the stress agents, the NCF cells were inherently more tolerant to all the stress agents than the SCF1 and SCF2 cells. In the subsequent experiments involving rifampicin and H_2_O_2_, SCF1, SCF2, and NCF cells were exposed to 25 μg/ml of rifampicin for 4 h or to 0.8 mM H_2_O_2_ for 60 min. The reason is that the mean percentage survival of the cells was about 25% for SCF1 & SCF2 cells and about 60% for NCF cells against rifampicin and about 32% for SCF1 & SCF2 cells and 50% for NCF cells (*n* = 16 sample sets of SCF1, SCF2, and NCF each) against H_2_O_2_ (Figures 4A,B and E,F, respectively). It was also observed that exposure of the cells to the different stress agents for short durations, of less than 30 min, did not show any lethality and hence were not considered for studying differential susceptibility. The change in the H_2_O_2_ concentration was negligible throughout the incubation period of 60 min and even upto 75 min monitored (Supplementary Figure S3O), ruling out the possibility of a decrease in H_2_O_2_ concentration that otherwise could have been the cause for the differences in the susceptibility of SCF1, SCF2, and NCF cells to the stress. Concentration of rifampicin also has not been found to be changing appreciably upon prolonged exposure to cells (data not shown).

### Live cell imaging confirms differential susceptibility of SCs and NCs to rifampicin

The differential susceptibility of SCs and NCs to rifampicin was confirmed using live cell imaging of the growth and division of sister daughter cells freshly born from normal/long-sized mother cells that divided by highly-deviated ACD to generate sister SC-NC pairs and short-sized and normal/long-sized mother cells that divided symmetrically to generate sister SC-SC pairs and sister NC-NC pairs, respectively. The readout on the differential rifampicin susceptibility of the sister daughter cells was the time taken by the sister daughter SC-NC, SC-SC, and NC-NC pairs to grow and divide post-exposure to the stress applied on the sister daughter cells immediately post-division of their respective mother cells and withdrawn later. However, following the exposure of the cells on agarose pad to 25 μg/ml rifampicin for 1 h (as it had been used in Figures 4A,B), no detectable growth of the cells was observed in the 8 h monitored, probably due to the high rifampicin to cell density ratio on the agarose pads, which might have become lethal, unlike in flask cultures. Therefore, exposure to 12.5 μg/ml of rifampicin for 50 min was used as the optimum condition.

**Figure 5:**
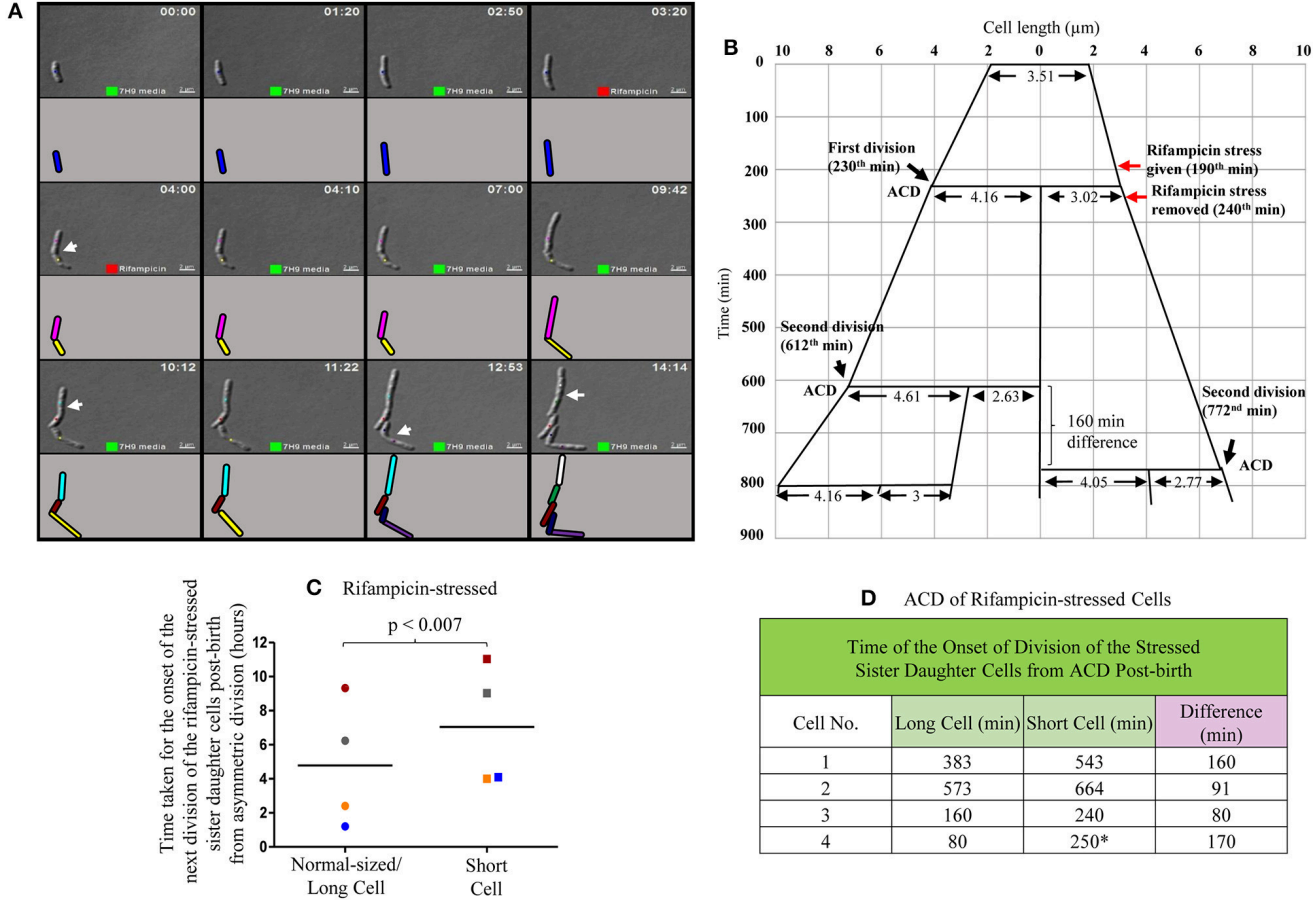
**Live cell imaging of *Msm* MLP cell undergoing ACD in the presence of rifampicin, its lineage and data on the time taken by the rifampicin-stressed sister daughter cells (post-birth) generated from ACD**. **(A)** Live cell imaging panels of *Msm* cells exposed to 12.5 μg/ml rifampicin for a period of 50 min. Rifampicin exposure was given for 50 min in agarose pad, *t* = 3:10-4:00 (red square), replaced with Middlebrook 7H9 medium after *t* = 4:00 and monitored for 10 h. Cell, marked with blue color, underwent asymmetric division generating a short daughter cell and a normal/long-sized daughter cell. The arrows show the division sites in the respective frames. Each cell is represented in different color and as the cell divided, the daughter cells have been given a different color in the cartoon representation. The panel **(A)** corresponds to the images in the Video S2. **(B)** The lineage of the growth and division of the rifampicin-stressed *Msm* cell undergoing ACD (shown in the panel **A**). **(C)** Time taken for the onset of the next division of rifampicin-treated sister daughter cells (normal/long-sized cell and short cell) generated from ACD, data shown in **(D)**. The sister cells have been marked in the same color, in **(C)**. The * sign in **(D)** indicates the ACD-generated short cell that never grew in the observed time period.

In the three out of four cases of highly-deviated ACD of normal/long-sized mother cells, the daughter SCs either never grew or took significantly longer time to undergo the next division as compared to the sister daughter NCs (Video S2; Figure 5A, Supplementary Figure S4). For example, the mother cell of length 3.51 μm was rifampicin-stressed prior to ACD and the rifampicin was removed post-ACD, which generated two sister daughter cells of lengths, 4.16 μm (NC) and 3.02 μm (SC), respectively (Figure 5B). While the NC grew and further divided in 382 min, the growth and division of the SC was further delayed by 160 min (Figure 5B). All the four sets of NC-SC pairs monitored showed statistically significant delay for the growth and division of rifampicin-stressed SC, as compared to the rifampicin-stressed NC (Figures 5C,D; *p* < 0.007; *n* = 4). In one of the four cases, the short sister daughter cell did not grow (even upto 250 min observed) upon exposure to rifampicin (Figure 5D, indicated by the * sign; Supplementary Figure S4A). More examples are given in Supplementary Figure S4. The differential susceptibility observed for the rifampicin-stressed sister daughter SC-NC pairs in the liquid medium present in the agarose pad correlated with the higher rifampicin-susceptibility of the SCs in the SCF over the NCs in the NCF observed in the liquid cultures in flasks, determined using cfu.

### Live cell imaging confirms differential susceptibility of SCs and NCs to H_2_O_2_

**Figure 6:**
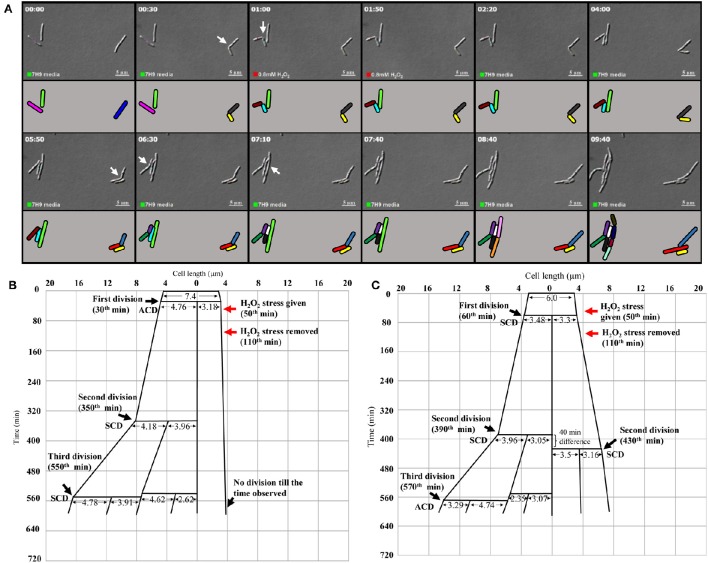
**Live cell imaging of *Msm* MLP cells undergoing ACD and SCD in the presence of H_2_O_2_ and their respective lineage. (A)** Live cell imaging panels of *Msm* cells exposed to 0.8 mM H_2_O_2_ for a period of 1 h, immediately after the first division and was replaced with Middlebrook 7H9 medium after 1 h of exposure. (ACD; the cell on the right side in the panels, SCD; the lower of the two cells on the left side in the panels). H_2_O_2_ was introduced at 50th min (indicated by the red square at the lower bottom of the panels) and was replaced with the medium at 1 h 50th min (indicated by the green square at the lower bottom of the panels). The treated cells were observed for about 9 h. The panel **(A)** corresponds to the images in the Video S3. **(B)** The lineage of the growth and division of the H_2_O_2_-stressed *Msm* cell undergoing ACD (shown in the right side of the panel **A**). **(C)** The lineage of the growth and division of the H_2_O_2_-stressed *Msm* cell undergoing SCD (shown in the left side of the panel **A**). The zero time point does not correlate with the birth of the starting mother cell. The cell lengths given are from the DIC images, but not drawn to scale. The time of generation of daughter cells from asymmetric cell division (ACD) and symmetric cell division (SCD) has been indicated for each division. The time point of exposure to the stress and its withdrawal have been indicated with red arrows.

Live cell imaging was performed to confirm the differential susceptibility of SCs and NCs to oxidative stress also. The H_2_O_2_-stressed sister daughter SC (3.18 μm, yellow), formed from the ACD of 7.4 μm long mother cell (the blue mother cell depicted in Figure 6A, on the right side in the panel), did not grow at all for the 560 min monitored (Figure 6B; Video S3; more images in Videos S4, S5). On the contrary, the H_2_O_2_-stressed sister daughter NC (4.76 μm), formed from the ACD of the same 7.4 μm long mother cell, grew and divided symmetrically to generate two daughter cells (4.18 and 3.96 μm) that further underwent SCD and ACD, respectively (Figure 6B). During the same time, in the vicinity of the same mother cell, the H_2_O_2_-stressed sister daughter cells of comparable sizes (3.48 and 3.30 μm), formed from the SCD of another mother cell of length 6.0 μm (the pink mother cell depicted in Figure 6A, on the left side in the panel), grew and divided by SCD (with only 40 min difference) to generate comparably-sized daughter cells, one of which further grew and divided by highly-deviated ACD while the other grew and divided by SCD to produce the next generation of daughter cells (Figure 6C). Thus, the H_2_O_2_-stressed SCs generated from highly-deviated ACD of normal/long-sized mother cells never grew or divided or took more time to grow and divide, than their normal/long-sized sister daughter cells.

More examples of the growth and division without delay of the sister daughter cells formed from SCD of H_2_O_2_-stressed normal/long-sized mother cells are given (Video S6, Supplementary Figures S5A,B). It was of interest to note that the H_2_O_2_-stressed sister daughter SCs arising from the SCD of short-sized mother cells always grew and divided approximately at the same time, if at all with minor time difference in the onset of the division (Supplementary Figures S5A,B). Sometimes, the H_2_O_2_-stressed short sister daughter cells (SCs formed from SCD) elongated together but at different rates but never divided in the period monitored (Supplementary Figure S5B). Also, such sister daughter SCs that grew and divided together (or grew together but did not divide) were always equally susceptible and not differentially susceptible to stress. Thus, the behavior of the H_2_O_2_-stressed sister daughter SCs, formed from SCD of short-sized mother cells, in growth and division were always comparable. The differential susceptibility observed for the H_2_O_2_-stressed sister daughter SC-NC pairs in the liquid medium present in the agarose pad correlated with the higher susceptibility of the SCs in the SCF over the NCs in the NCF observed in the liquid cultures in flasks, determined using cfu.

### Quantitative comparison of the effect of H_2_O_2_ stress on sister daughter SCs and NCs

**Figure 7:**
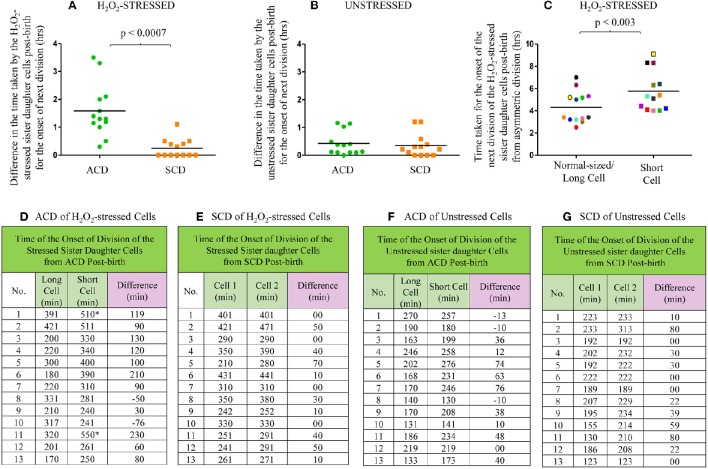
**Live cell imaging data on the time taken by the H_2_O_2_-stressed or unstressed sister daughter cells (post-birth) generated from ACD and SCD. (A–C)** Time taken in hours or **(D–G)** in minute. **(A)** Difference in the time taken for the onset of next division by H_2_O_2_-treated sister daughter cells (post- birth) generated from ACD and SCD, data for ACD shown in **(D)** and data for SCD shown in **(E)**. **(B)** Difference in the time taken for the onset of next division by unstressed sister daughter cells (post- birth) generated from ACD and SCD, data for ACD shown in **(F)** and data for SCD shown in **(G)**. **(C)** Time taken for the onset of the next division of H_2_O_2_-treated sister daughter cells (normal/long-sized cell and short cell) generated from ACD, data shown in **(D)**. The sister cells have been marked in the same color, in **(C)**. The * sign in **(D)** indicates the two ACD-generated short cells that never grew in the 8 h examined. The minus sign in **(D)** indicates the two ACD-generated short cells that grew earlier than the respective sister normal/long-sized cell. The minus sign in **(F)** indicates the three ACD-generated short cells that grew earlier than the respective sister normal/long-sized cell, *n* = 13 independent samples.

From the live cell images of several samples, the time taken by the H_2_O_2_-stressed sister daughter cells for the onset of the first division post-birth from highly-deviated ACD and SCD were determined. For the onset of the first division post-birth, the differences in the time taken by the H_2_O_2_-stressed sister daughter cells from highly-deviated ACDs were significantly higher than the differences in the time taken by the H_2_O_2_-stressed sister daughter cells from SCDs (Figures 7A,D,E; *p* < 0.0007; *n* = 13). The sister daughter SCs showed significant delay in growth and division in 9/13 cases (Figures 7A,D) and no growth and division in 2/13 cases in the period monitored (Figures 7A,D, indicated by the * sign). However, it was of interest to note that in the remaining 2/13 cases, the stressed sister SC grew earlier than the stressed sister NC (Figures 7A,D, indicated by the minus sign). At present, we do not have an explanation for this deviation. It may be recalled here that when the Percoll fractions were exposed to H_2_O_2_, in 1–10% of the cases, SCF1 and SCF2 cells showed higher survival than NCF cells (see Figure 4F and Supplementary Figures S3B,D,H). Thus, the deviation in the oxidative stress response of the 1–10% of the SC population was comparable in liquid culture (determined using cfu) and on agarose pads (during live cell imaging).

On the contrary, there was no significant difference between the time taken by the H_2_O_2_-stressed sister daughter cells (formed from SCD) for the onset of growth and next division (Figures 7A,E). Similarly, among the unstressed cells (control sample), there was no significant delay between the sister daughter cells, formed either from highly-deviated ACD or SCD, for the growth and onset of next division post-birth (Figures 7B,F,G). Further, between the H_2_O_2_-stressed sister daughter SCs and NCs, post-birth from highly-deviated ACD, the time taken for the growth and onset of next division was significantly (*p* < 0.003) more for the sister daughter SCs than that for the sister daughter NCs (Figures 7C,D). However, in the 3/13 unstressed samples, the sister daughter SCs divided earlier than the sister daughter NCs (Figure 7F, indicated by the minus sign). Thus, live cell imaging of H_2_O_2_-stressed sister daughter cells formed from highly-deviated ACD confirmed that the sister daughter SCs were generally more susceptible to the stress than the sister daughter NCs. On the contrary, the stress tolerance of the SC-SC and NC-NC pairs formed from the SCD of short or normal/long-sized mother cells, respectively, was comparable.

### *Mtb* SCF cells are also significantly more susceptible to H_2_O_2_ than *Mtb* NCF cells

**Figure 8:**
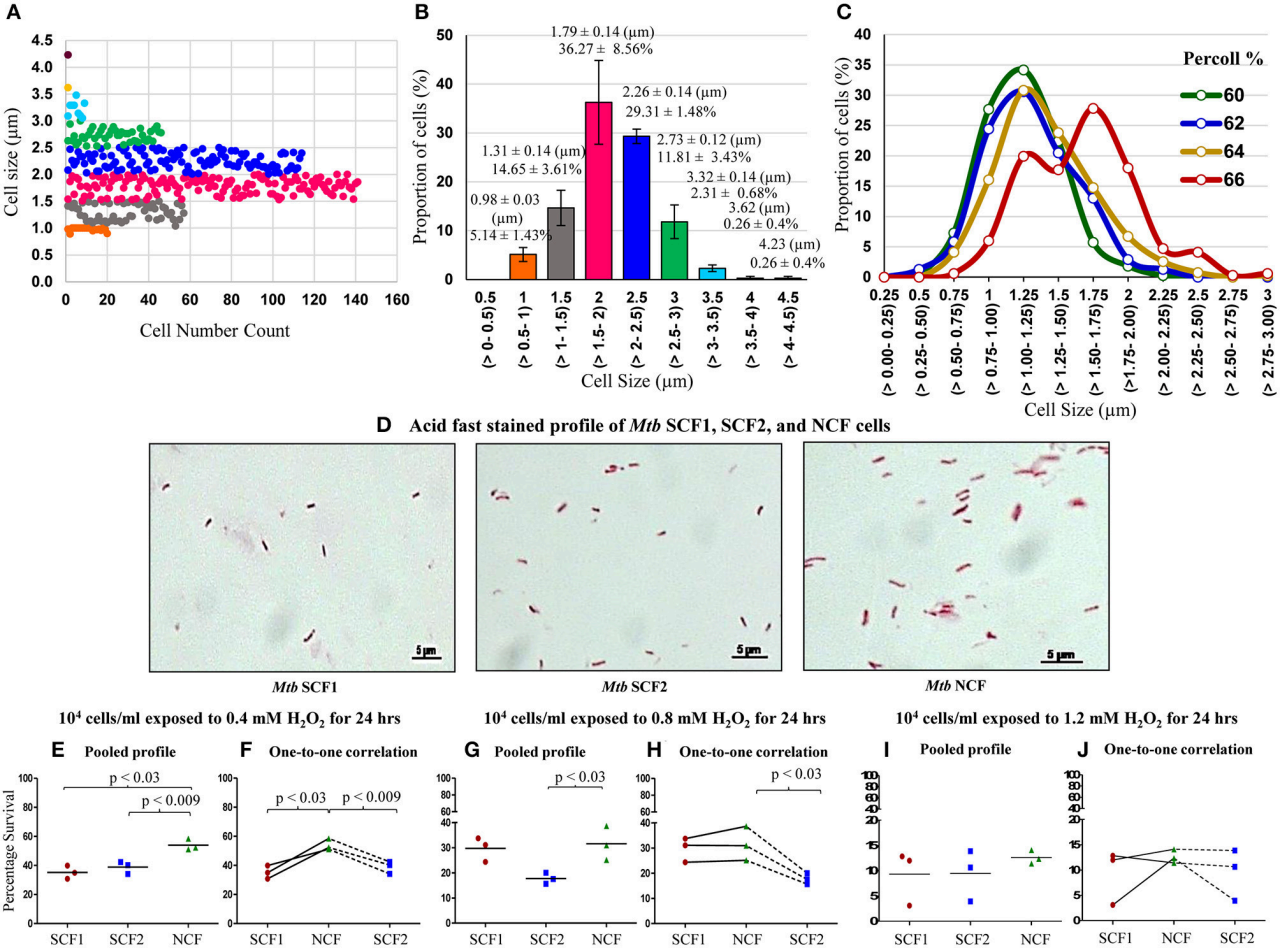
**Profile of cell-size-based subpopulations of *Mtb* from MLP, Percoll gradient fractions and susceptibility of the fractions to H_2_O_2_**. **(A)** Different subpopulations of cells with specific range of cell lengths. Each dot represents one cell (*n* = 389). **(B)** Quantitation of the proportion of the cells in each subpopulation shown in **(A)** (*n* = 3). **(C)** Proportion of cells is plotted against the *Mtb* cells in each length range from 60, 62, 64, and 66% Percoll fractions (*n* ≥ 300 cells counted from each Percoll fraction). The values in brackets show the range of the lengths indicated on the X-axis. **(D)** Profile of the composition of acid fast positive *Mtb* SCF1, SCF2, and NCF cells. Percentage survival of *Mtb* SCF1, SCF2, and NCF cells when exposed to: **(E,F)** 0.4 mM H_2_O_2_; **(G,H)** 0.8 mM H_2_O_2_ and **(I,J)** 1.2 mM H_2_O_2_ for 24 h (*n* = 3 samples each). **(E,G,I)** Pooled results of all the experimental sets; **(F,H,J)** Relative one-to-one correlation of the survival of the samples in each independent set.

Like in the case of *Msm* MLP population, Middlebrook 7H9 medium grown MLP cultures of *M. tuberculosis* (*Mtb*) also contained distinct sub-populations of cells that could be grouped unbiased into specific ranges of length (Figures 8A,B). Two sub-populations (~5 and 14%) of SCs in the size ranges of 0.5–1.0 and 1.0–1.5 μm, respectively, were present (Figures 8A,B, orange & gray). The NCs constituted major proportion (~65%) of the cells with a size range of 1.5–2.5 μm (Figures 8A,B, pink & dark blue). Low proportion of cells in the size range of 2.5–3.0 μm were also present (Figures 8A,B, green). In addition, very low proportions of cells longer than 3.0 μm also could be observed in the population (Figures 8A,B, light blue, yellow, & brown). These observations show the high level of cell size heterogeneity in the *Mtb* population.

Like in the case of *Msm* cells, fractionation of *Mtb* MLP cells on Percoll gradient showed a bimodal distribution in terms of cell length (Figure 8C; Supplementary Table S6). The average lengths of the *Mtb* cells in the low density Percoll fractions (60+62% & 64%) were comparable to that of the SCs from the earlier measurements of *Mtb* cells in MLP population (see Figures 8A,B; Supplementary Table S6). Since the average cell size of *Mtb* cells in 60% (1.12 ± 0.27 μm) and 62% (1.17 ± 0.32 μm) Percoll fractions were comparable, they were mixed together (1.14 ± 0.29 μm) and termed as *Mtb* SCF1 (Figures 8C,D; Supplementary Tables S6, S7). Similarly, the *Mtb* 64% Percoll fraction, which contained cells with average size (1.29 ± 0.35 μm) slightly higher than that of the *Mtb* SCF1 cells, was termed *Mtb* SCF2 (Figures 8C,D; Supplementary Table S7). Since mycobacterial cells of low buoyant density fractions in Percoll gradient have been found to be rich in lipid (Deb et al., 2009), the fractionation of SCs into low buoyant density Percoll fractions indicates high lipid content in SCF cells, as in the case of *Msm* SCF cells.

The majority (~70%) of *Mtb* cells in the high density 66% Percoll fraction showed an average length (1.54 ± 0.39 μm) comparable to that of the NCs from the earlier measurements of cells in MLP population (see Figures 8A,B; Supplementary Tables S6, S7). Therefore, the 66% fraction of *Mtb* cells was called **n**ormal-sized **c**ells' **f**raction (*Mtb* NCF) (Figures 8C,D; Supplementary Table S7). Interestingly, the 66% NCF showed a partially bimodal distribution of cell size (Figure 8C), probably due to the high level of cell size heterogeneity among the cells that have higher density. The *Mtb* SCF1, SCF2, and NCF cells also contained outliers, which were cells having lengths much higher (in SCF1 & SCF2) or much lower (in NCF) than the average length of the cells in the respective fraction, but having same buoyant density. These cells were also included in the determination of average length of the cells in the respective Percoll fraction (Figures 8C,D; Supplementary Table S7). The 68–76% *Mtb* Percoll fractions contained very few or no cells (Supplementary Table S6).

The *Mtb* SCF1 and SCF2 cells showed significantly low percentage survival than *Mtb* NCF cells against a range of H_2_O_2_ concentrations (0.4–1.2 mM) exposed for 24 h (Figures 8E–J). Thus, in spite of being a pathogenic species, the heterogeneity in the *Mtb* population was maintained in a manner similar to its saprophytic counterpart such that there exists two sub-populations, the significantly more H_2_O_2_-susceptible SCF cells and the H_2_O_2_-tolerant NCF cells. This alluded to the possibility that the cell size/density heterogeneity and the differential stress susceptibility thereof are not influenced by the pathogenic status of the bacterium. Thus, the maintenance of cell size/density heterogeneity and the consequential differential stress susceptibility seem to be the inherent physiological property of mycobacteria.

## Discussion

### Cell size and cell density based heterogeneity in mycobacterial populations

Our studies show that *Msm* and *Mtb* MLP populations naturally harbor a very high level of heterogeneity, with the two sub-populations of SCs and NCs, standing out in terms of their difference in size, density, and uniform differential susceptibility to three prominent stress conditions, the antibiotic, oxidative and nitrite stress that are faced by mycobacteria in their natural habitats. The generation of SCs through the ACD of NCs and SCD of SCs in the nutrient-rich actively growing MLP population ruled out reductive cell division (without growth and elongation of mother cells) for their generation as it occurs only in mycobacterial cultures that approach stationary phase (Smeulders et al., 1999 and unpublished observations from our laboratory). Another possibility for the generation of SCs is slow growth of NCs followed by division before the mother cell attains double the birth size through growth and elongation. This could be a possibility as mycobacteria are known to divide exactly based on time after one round of DNA replication (Hiriyanna and Ramakrishnan, 1986) and not on cell size attainment, unlike *E. coli* (Løbner-Olesen et al., 1989). However, in the live cell imaging, we did not see slow growth followed by division before the mother cell attained double the birth size in the samples monitored (*n* = 10 mother cells).

The partitioning of the SCs into low density Percoll fractions, indicative of higher lipid content, correlated with the large number of MVs present on their surface, as MVs are known to be rich in lipid content (Prados-Rosales et al., 2011). Conversely, the partitioning of NCs into the high density Percoll fractions also correlates with the negligible number of MVs on their surface. Since the cellular properties of the SCs and the NCs were not prior known to us, the only way they could be fractionated was based on the differences in their size. Although centrifugal elutriation could have been used for their size-based fractionation, we resorted to Percoll gradient centrifugation as mycobacterial cells and spores have earlier been fractionated using Percoll gradient centrifugation (Deb et al., 2009; Ghosh et al., 2009). Thus, it is possible that serendipitously we have fractionated the *Msm* and the *Mtb* MLP populations based on cell density differences although our intention was to fractionate them based on size differences. The cell density based fractionation of the SCs and the NCs enabled us to study their differential stress susceptibility truly based on cell density differences that were probably indicative of differences in their metabolic status controlled by multiple molecular mechanisms.

The stable maintenance of ~10% of SCs and ~90% of NCs in the *Msm* and *Mtb* MLP cultures irrespective of the growth media indicated that the proportions of the sub-populations of SCs and NCs were not influenced by different growth media. It further implied that the stable maintenance of the SCs and the NCs in the specific proportions may be an inherent feature of mycobacterial populations and that the bacilli might be achieving it through cell division regulation which needs further investigation. The processes of centrifugation for pelleting the cells from the culture, suspension and washing of the cells with PBS followed by centrifugation, and final suspension of the cells in paraformaldehyde, altogether spanned for about 30 min out of the 3 h duration of the generation time of *Msm* cells and 24 h duration of the generation time of *Mtb* cells. However, these processes did not affect the cell size distribution as the unfixed cells taken directly from the culture and examined under DIC microscopy also showed similar size distribution with a difference of ~2–7% as compared to the respective proportions of SCs and NCs in the processed samples, which was not statistically significant (data not shown).

### Unique morphological and physiological features of SCs and NCs

**Figure 9:**
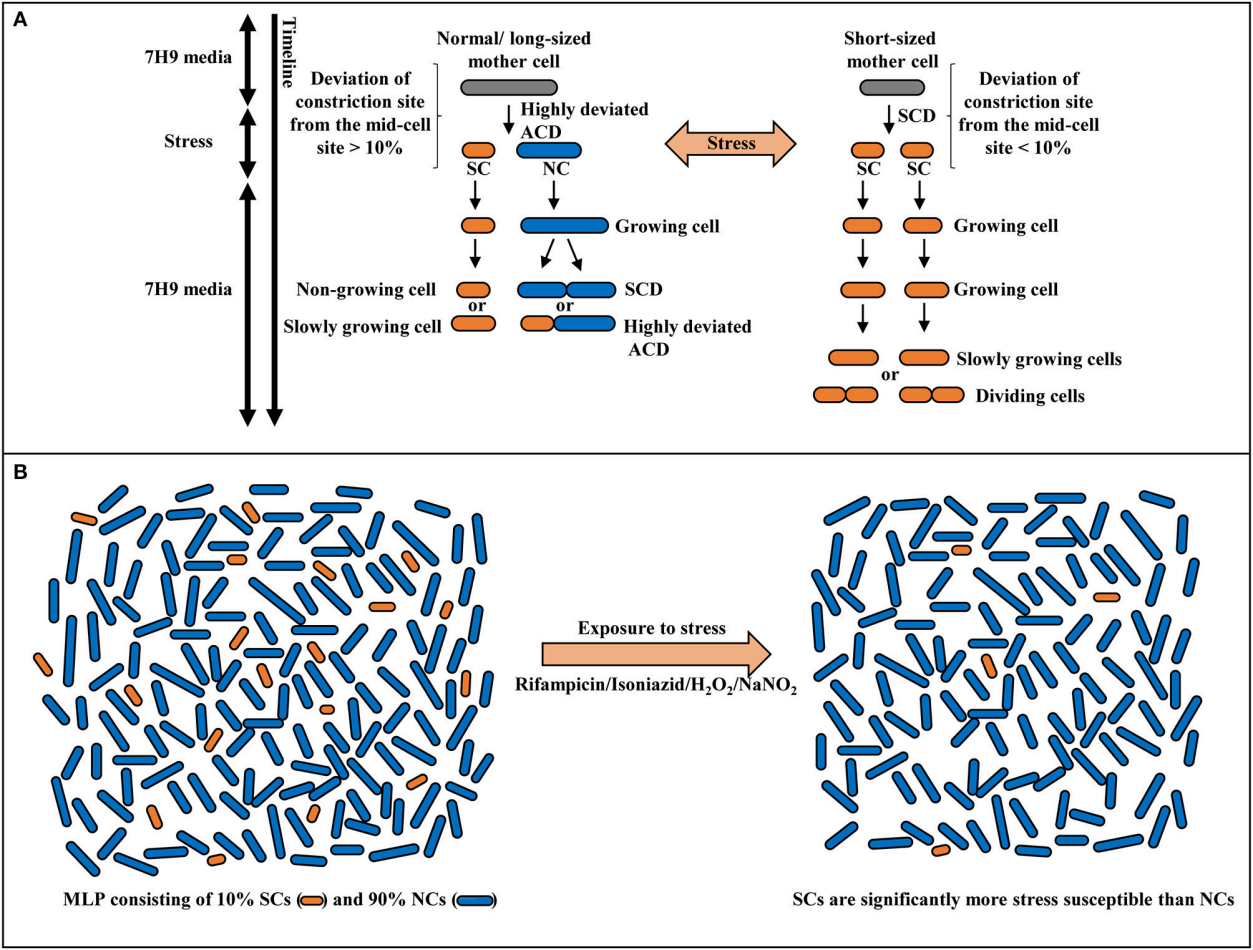
**Model for the stress response of *Msm* mid-log phase short cells (SCs) and normal-sized cells (NCs). (A)** Differential susceptibility of SCs and NCs generated from highly-deviated ACD of normal/long-sized mother cell and comparable susceptibility/ tolerance of sister daughter cells formed from SCD of short-sized mother cells, on exposure to stress. **(B)** Survival of SCs and NCs in the mid-log phase population (MLP) on exposure to antibiotic, oxidative or nitrite stress conditions. The SCs exhibit significantly more susceptibility to stress in comparison to NCs.

The morphological and physiological properties and stress susceptibilities of the sub-populations of *Msm* and *Mtb* SCs and NCs revealed several unique features, which made them distinct from the short-sized or normal-sized cells found under extreme stress conditions reported earlier in both pathogenic and non-pathogenic mycobacteria (Nyka, 1974; Khomenko, 1987; Smeulders et al., 1999; Thanky et al., 2007; Aldridge et al., 2012; Richardson et al., 2016; Wu et al., 2016). (i). the presence of SCs in the mid-log phase population in nutrient-rich medium suggested that their presence is not stress-condition dependent unlike the short-sized cells found during late stationary phase in mycobacteria (Smeulders et al., 1999), or ovoid form of mycobacteria found under extreme starvation (Nyka, 1974; Anuchin et al., 2009) or the cell-wall-deficient L-forms of mycobacteria (Imaeda, 1975; Korsak, 1975; Markova et al., 2012); (ii). the fact that they form specific sub-populations, the proportions of which do not change in different growth media, indicated some specific functional role for the SCs and the NCs in the mycobacterial population; (iii). the low density of SCs in the SCF, probably due to the presence of large number of MVs (known to be rich in lipids and used by bacteria to acquire molecules deficient in the cell or supply of molecules deficient in other sub-populations; Prados-Rosales et al., 2014) on their surface, unlike the high density of NCs in the NCF that have negligible number of MVs on the surface, indicated some distinct role for them in stress responses involving specific molecules (Vaubourgeix et al., 2015); (iv). the acid-fast stainability of SCs and NCs indicated their typical mycobacterial characteristics and that they are unlike the chromophobic cells starved on plain agar (Nyka, 1974); (v). the heat-susceptibility showed that the SCs and the NCs are not spores that are found only in highly aged cultures of *M. smegmatis, M. marinum, M. bovis* (Ghosh et al., 2009), and *M. avium* Subsp. *paratuberculosis* (Lamont et al., 2012); (vi). the culturability of SCs in liquid and solid media indicated the full genome content and population regeneration potential of SCs and that they are complete mycobacterial cells like the NCs and that they were neither VBNC (viable-but-not-culturable) cells (Shleeva et al., 2002, 2004), nor like the short anucleated *M. smegmatis* cells generated upon overexpression of *M. tuberculosis* ParB (Maloney et al., 2009) nor like the DNA-free mini cells arising due to aberrant division reported in *Escherichia coli* (Adler et al., 1967); (vii). the uniform susceptibility of SCs to antibiotic, oxidative, and nitrite stress, unlike the NCs, in spite of the comparable duration of their growth and division under stress-free conditions and slow growth or lack of further growth of SCs under stress, showed distinct metabolic status of the two types of sub-populations indicative of some specific physiological purpose. These features of the SCs and the NCs are depicted in a model (Figure 9).

### Does the differential stress susceptibility of SCs and NCs correlate with their differential cell density?

The conspicuous partitioning of the stress-susceptible SCs into low density Percoll fractions (SCF1 and SCF2; 0.936 and 1.023 gm/ml, respectively) and of the stress-tolerant NCs into high density Percoll fractions (1.061 g/ml) may be indicative of a correlation of differential cell density to differential stress susceptibility. It has been found that multiple-stressed mycobacterial cells having higher lipid content fractionate into low buoyant density Percoll fractions (Deb et al., 2009) and further the MVs found on the surface of mycobacterial cells are rich in lipid content (Prados-Rosales et al., 2011). Based on these reports and from our finding that the SCs in the low density SCF have large number of lipid-rich MVs on their surface, unlike the high density NCF cells with negligible MVs on their surface, it is tempting to speculate that the high lipid content indicated by the low density may be one of the reasons for the significantly higher stress-susceptibility of the SCs and lower susceptibility of the NCs. Thus, the correlation of short cell size with higher stress susceptibility and normal/long size with stress tolerance might have been just incidental. It implies that the very low proportions of normal/longer-sized cells (arising probably from long-sized mother cells) in the low density stress-susceptible SCF cells also may have high lipid content, while the very low proportions of short-sized cells (probably arising from the short-sized mother cells) in the high density stress-tolerant NCF cells also may have low lipid content.

*M. tuberculosis* cells exposed to H_2_O_2_ were reported to contain significant levels of lipid hydroperoxide as compared to the untreated bacilli (Cirillo et al., 2009). Possibilities of oxidative lipid damage have also been reported during the exposure of the bacterial cells to antibiotics (Belenky et al., 2015). Lipid peroxidation triggers a free radical chain process which results in the formation of several reactive oxygen species (Slater, 1984). It has also been reported that further oxidation of oxidized phospholipids is a stochastic process and results in the formation of a wide range of oxidized phospholipids (Bochkov et al., 2010). Thus, the severity of lipid peroxidation might depend on the effects of different products formed during the process. Lipid hydroperoxides are highly unstable and they decompose to give different products, of which malondialdehyde is an important product (Marnett, 1999) that leads to inter-strand cross-link formation in DNA, resulting in mutagenesis and lethality in *E. coli* (Yonei and Furui, 1981). Further, lipid peroxidation in *E. coli* has also been found to result in the loss of membrane integrity, thereby affecting their survival (Joshi et al., 2011). Moreover, mycobacterial cells having higher lipid content fractionated into low buoyant density regions subsequent to Percoll density gradient centrifugation (Deb et al., 2009) and mycobacterial MVs were found to be rich in lipid (Prados-Rosales et al., 2011). The presence of high density of MVs on SCs and low density of MVs on NCs and their fractionation into low density Percoll fractions and high density Percoll fractions, respectively, were suggestive of high levels of lipid in SCs and low levels in NCs. All these observations tempted us to speculate that one of the causes for the higher susceptibility of SCs might be lipid peroxidation associated damages to the cells.

### Extent and differential survival of SCs and NCs against the stress conditions are robust but stochastic

The significantly higher survival of the NCs than the SCs, when exposed to a range of concentrations of rifampicin, isoniazid and H_2_O_2_ for various durations, showed the robustness of the survival response against these stress conditions. The mean percentage of survival below the lowest value in the range of rifampicin, isoniazid, and H_2_O_2_ concentrations studied was more than 90% and that above the highest range examined was less than 10%. Hence the studies were restricted to the indicated concentrations only. The processes involved in the survival of the SCF and NCF cells and of the higher survival of NCF cells over the SCF cells may be stochastic in nature since the extent of survival of the SCF and NCF cells individually and of the higher survival of NCF cells than the SCF cells against each of the different stress conditions was not the same from individual to individual experiments. In spite of such stochasticity, the mean percentage survival of NCF cells, as compared to that of SCF1 and SCF2 cells, always remained higher, consistent, reproducible, and statistically significant against each of the stress condition at different cell densities and different durations of exposure. Only in about 1–10% cases, the SCF cells showed higher survival than the NCF cells. Further, while the sister daughter SC-NC pairs, which arise from the highly-deviated ACD of NCs, showed differential stress-susceptibility, the SC-SC pairs generated from the SCD of SCs invariably showed comparable susceptibility/tolerance to stress, like the NC-NC pairs from the SCD of NCs. While this is the case in the stress-susceptibility difference in the individual pairs (SC-NC or SC-SC) of sister daughter cells, the total population of SCs (generated from the highly-deviated ACD of NCs and SCD of SCs) in the SCF1 and SCF2 were more susceptible than the NCs in the NCF.

The observations indicating the existence of a certain level of inherent stochasticity in the extent of survival of the SCF1, SCF2, and NCF cells against rifampicin, isoniazid, H_2_O_2_ and acidified nitrite tempted us to speculate on the possible reasons for the phenomenon. It has been well documented that the survival against H_2_O_2_ is mediated by the catalase-peroxidase enzyme (KatG) (Loprasert et al., 1988; Loewen and Stauffer, 1990; Heym et al., 1993) that dismutates H_2_O_2_ into dioxygen and water (Vlasits et al., 2007). The response of mycobacteria against nitrite stress also has been found to be similar to that against oxidative stress (Voskuil et al., 2011). It is also known that isoniazid activation is dependent on KatG (Shoeb et al., 1985; Johnsson and Schultz, 1994), which is required for the susceptibility of mycobacterial cells to isoniazid (Cohn et al., 1954; Middlebrook et al., 1954; Zhang et al., 1992) and other antibiotics also (Nandakumar et al., 2014). Further, atleast in the case of isoniazid activation, it has been established that the *katG* promoter, which drives the expression of *katG* gene, is stochastically active (Wakamoto et al., 2013). Thus, it is possible that the stochasticity in the KatG-dependent survival of the SCF and NCF cells against H_2_O_2_, acidified nitrite, rifampicin and susceptibility to isoniazid might be reflective of the stochastic synthesis of KatG in these cells. The fact that the proportion of SCs and NCs were maintained stably and that the SCs were always found to be significantly more stress-susceptible than the NCs showed that the cells were not unstable and were responding consistently and that the phenomenon is not a random one. Thus, the stochasticity in the extent of survival/susceptibility of individual SCs and NCs and of the higher survivability of NCs over SCs, may be indicative of the stochasticity of the processes involved rather than the possibility of instability of the cells themselves.

### Asymmetry in daughter cells' fate in mycobacteria, other bacteria, and other living systems

Sister daughter cells undergoing different fates post cell division in response to stress, indicative of metabolic differences between them, has been known in many biological systems, including bacteria. In order to propagate the progeny, *Caulobacter crescentus* generate differently-fated stalked cells and the swarmer cells during cell division (Tsokos and Laub, 2012). A subpopulation of the *Cryptococcus gattii* fungal cells, with tubular mitochondrial morphology attained in response to host reactive oxygen species, were found to lose their proliferative ability, as compared to their kin with non-tubular mitochondrial morphology (Voelz et al., 2014). The generation of metabolic asymmetry in daughter cells, causing different fates to sister daughter cells, is known to occur in alpha-proteobacteria, *Volvox carteri* (alga), *Drosophila* (fly), *Caenorhabditis elegans* (worm), and in mouse (reviewed in Inaba and Yamashita, 2012). Thus, the generation of asymmetry in the fates of sister daughter cells seems to be a conserved strategy in the living kingdom to achieve specific physiological advantages in specific organisms under different growth, developmental, and stress conditions. In mycobacteria, asymmetric partitioning of the irreversibly oxidized proteins (IOPs), resulting in the differential accumulation of these IOPs between the sister-daughter cells, has been reported (Vaubourgeix et al., 2015). The sister-daughter cell that has sequestered more IOPs has been found to become more prone to stress conditions (Vaubourgeix et al., 2015). In line with these observations, it may be possible that during the asymmetric division of the mycobacterial mother cell, a differential partitioning of specific protein(s) or other molecular factor(s) may occur, which may confer differential susceptibility to the sister daughter cells to specific stress agents.

## Author contributions

PA, SV, RN, DS, and KJ conceived/designed expts; SV, RN, DS, KJ, NM, and AP performed expts; PA, SV, RN, DS, NM, SS, and NJ analyzed data; NJ did statistical analysis; PA contributed reagents, materials, and analysis tools; PA, SV, and RN wrote the manuscript. SV and RN contributed equally to the work. All the authors have read and approved the manuscript.

### Conflict of interest statement

The authors declare that the research was conducted in the absence of any commercial or financial relationships that could be construed as a potential conflict of interest.
