# Supplementary material for: Mycobacterial Cultures Contain Cell Size and Density Specific Sub-populations of Cells with Significant Differential Susceptibility to Antibiotics, Oxidative and Nitrite Stress
Source: Front Microbiol. 2017 Mar 21;8:463. doi: 10.3389/fmicb.2017.00463 (PMC5359288; doi:10.3389/fmicb.2017.00463)
Supplement: Supplementary file 7 [file Presentation1.pdf]

Supplementary Material

Mycobacterial Cultures Contain Cell Size and Density Specific Sub-populations of Cells with Significant Differential Susceptibility to Antibiotics, Oxidative and Nitrite Stress

Srinivasan Vijay, Rashmi Ravindran Nair, Deepti Sharan, Kishor Jakkala, Nagaraja Mukkayyan, Sharmada Swaminath, Atul Pradhan, Niranjan V. Joshi, Parthasarathi Ajitkumar*

*Corresponding author:

Parthasarathi Ajitkumar,
Tel: 91-80-2293-2344; Fax: 91-80-2360-2697;
E-mail: ajitkpartha@gmail.com

This document contains:

- Supplementary Data
- Supplementary Tables (S1-S7)
- Supplementary Text
- Supplementary Figures (S1-S5)
- Supplementary Video Legends (Video S1 - Video S6)

SUPPLEMENTARY DATA

The Composition and Annotation of Different *Msm* and *Mtb* Percoll Fractions

The cells were fractionated using Percoll gradient centrifugation (**Figures 1E,F; Supplementary Table S3**). The cell lengths were measured under bright field after acid fast staining or under DIC and the average cell sizes ± SD were obtained for cells from each fraction. The 64% and 66% *Msm* Percoll fractions obtained from the analytical scale Percoll gradient fractionation, were mostly containing shorter cells (2.34 ± 0.65 µm and 2.52 ± 0.76 µm, respectively) and were termed as *Msm* **S**hort-sized **C**ells' enriched **F**raction 1 (*Msm* SCF1) and *Msm* **S**hort-sized **C**ells' enriched **F**raction 2 (*Msm* SCF2) (**Supplementary Tables S3, S4**) based on the proportion of shorter cells in these fractions. The 68-76% and 80% Percoll fractions of *Msm* cultures contained cells of average cell size increasing from 2.89 ± 0.91 to 3.53 ± 0.98 (**Supplementary Table S3**). These were combinedly called the *Msm* **M**ixed-sized **C**ells' **F**raction (*Msm* MCF) (**Supplementary Table S3**). Whereas, the 78% Percoll fraction alone contained cells having higher size range (3.71 ± 0.98 µm) and hence it was termed *Msm* **N**ormal-sized **C**ells' enriched **F**raction (*Msm* NCF) (**Supplementary Tables S3, S4**). The average sizes of *Msm* cells in the 64% (2.34 ± 0.65 µm), 66% (2.52 ± 0.76 µm), and 78% (3.71 ± 0.98 µm) Percoll fractions obtained from the analytical scale Percoll gradient fractionation were comparable to the respective fractions of the preparative scale Percoll gradient fractionated *Msm* cells in 64% (SCF1, 2.38 ± 0.59 µm), 66% (SCF2, 2.53 ± 0.64 µm) and 78% (NCF, 3.64 ± 1.03 µm) (compare **Supplementary Tables S3, S4** with **Supplementary Table S5**). Although 70-80% of *Msm* cells in the SCF1, SCF2 and NCF were of average size ± SD, 20-30% of the cells were outliers whose sizes were above or below the average size ± SD (**Supplementary Table S4**).

In the case of *Mtb* cells, the proportion of short-sized cells was found to be higher in the 60%, 62%, and 64% Percoll fractions. Since the average cell size of *Mtb* cells in 60% (1.12 ± 0.27 µm) and 62% (1.17 ± 0.32 µm) Percoll fractions were comparable, they were mixed together (1.14 ± 0.29 µm) and termed as *Mtb* SCF1 (**Supplementary Tables S6 and S7**). Subsequently, the *Mtb* 64% Percoll fraction, which contained cells with average size (1.29 ± 0.35 µm) slightly higher than the *Mtb* SCF1, was termed *Mtb* SCF2 (**Supplementary Tables S6 and S7**). The 66% Percoll fraction contained higher proportion of normal-sized/ longer cells (1.54 ± 0.39 µm) and was termed *Mtb* NCF (**Supplementary Tables S6 and S7**). The 68-76% *Mtb* Percoll fractions contained very few or no cells. Although 70-80% of *Mtb* cells in the SCF1, SCF2 and NCF were of average size ± SD, 20-30% of the cells were outliers whose sizes were above or below the average size ± SD (**Supplementary Table S7**).

Supplementary Table S1. List of bacterial strains used in the study

Name	Reference or Source
**Bacterial strains**	
Mycobacterium smegmatis mc^2155	William R. Jacobs (Snapper et al., 1990)
Mycobacterium tuberculosis $H_{37}R_a$	JALMA Institute of Leprosy and Other Mycobacterial Diseases, Agra, India

Supplementary Table S2. Composition of SCs and NCs in different culture media

Culture Medium	No. of SCs	No. of NCs	Total No. of cells counted	Proportion of SCs (%)	Proportion of NCs (%)
Youmans and Karlson's Medium	38	273	311	12.23 ± 1.86	87.77 ± 1.86
Dubos Broth Base	39	262	301	12.96 ± 3.24	87.04 ± 3.24
Sauton's Medium	60	302	362	16.57 ± 1.22	83.43 ± 1.22

Supplementary Table S3. The average length ranges of *Msm* cells from each percoll fraction, as measured under bright-field, post acid-fast staining

Percoll fractions (% of percoll)	Name of Percoll fraction SCF/ MCF/ NCF	*M. smegmatis* (*Msm)* Cells Average cell size (μm) from each percoll fraction (n ≥ 300)
64	SCF1	2.34 ± 0.65
66	SCF2	2.52 ± 0.76
68	MCF	2.89 ± 0.91
70	MCF	3.38 ± 1.06
72	MCF	3.41 ± 0.98
74	MCF	3.50 ± 0.91
76	MCF	3.50 ± 0.96
78	NCF	3.71 ± 0.98
80	MCF	3.53 ± 0.98

The 64% and 66% percoll fractions were mostly containing SCs and hence they were termed **s**hort-sized **c**ells' **f**raction 1 & 2 (SCF1 & SCF2) based on their frequency of length distribution (**Figure 1E,F**), respectively. The 78% percoll fraction was mostly composed of NCs and was called **n**ormal-sized **c**ells' **f**raction (NCF). All the other percoll fractions of *Msm* cells were called **m**ixed-sized **c**ell **f**ractions (MCF).

Supplementary Table S4. The average size ranges (± SD) and the proportions of *Msm* cells from analytical scale percoll gradient fractionated SCF1, SCF2, NCF, and of the outliers in each fraction, as measured under bright-field (BF), post acid-fast staining (n ≥ 300 cells) or under DIC

Average cell length / Proportion Measurements of *Msm* Cells from Analytical Scale Percoll Fractionation [Length Measured Under Bright-field (BF) or DIC]			
Name and % of the Percoll Fraction	Length (µm) Average ± SD	Proportion	Proportion of Outliers
SCF1 (64% Percoll)	2.34 ± 0.65 (BF)	77.55%	22.45%
	2.58 ± 0.71 (DIC)	69.04%	30.96%
SCF2 (66% Percoll)	2.52 ± 0.76 (BF)	75.35%	24.65%
	2.73 ± 0.90 (DIC)	73.60%	26.40%
NCF (78% Percoll)	3.71 ± 0.98 (BF)	68.33%	31.67%
	3.58 ± 1.16 (DIC)	72.99%	27.01%

The lines in the first column indicate the percoll fractions; the average lengths and SD of cells in the SCF1, SCF2, and NCF fractions as measured under bright-field (BF) (top lines) and under DIC (bottom lines) are indicated in the second column; their respective proportions are mentioned in the third column; the proportion of outliers which are much above or below the average length ± SD is indicated in the fourth column. These are cells, which were also counted to determine the average length, but having lengths much lower or much higher than the average length of the cells in the respective fraction, in spite of having same density as that of average sized cells. n ≥ 300 cells from each fraction.

Supplementary Table S5. The average size ranges (± SD) and proportions of *Msm* cells from preparative scale percoll gradient fractionated SCF1, SCF2, NCF, and of the outliers in each fraction, as measured under bright-field (BF), post acid-fast staining (n ≥ 300 cells)

Average Cell Length / Proportion Measurements of *Msm* Cells from Preparative scale Percoll Fractionation [Length as measured under BF]			
Name and % of the Percoll Fraction	Length (μm) Average ± SD	Proportion	Proportion of Outliers
SCF1 (64% Percoll)	2.38 ± 0.59	74.08%	25.92%
SCF2 (66% Percoll)	2.53 ± 0.64	75.07%	24.93%
NCF (78% Percoll)	3.64 ± 1.03	70.79%	29.21%

The lines in the first column indicate the percoll fractions; the average lengths and SD of cells in the SCF1, SCF2, and NCF fractions as measured under bright-field (BF) are indicated in the second column; their respective proportions are mentioned in the third column; the proportion of outliers which are much above or below the average length ± SD is indicated in the fourth column. These are cells, which were also counted to determine the average length, but having lengths much lower or much higher than the average length of the cells in the respective fraction, in spite of having same density as that of average sized cells.

Supplementary Table S6. The average size ranges of *Mtb* cells from each percoll fraction, as measured under bright-field, post acid-fast staining

Percoll Fractions	*M. tuberculosis (Mtb)*
(% of Percoll)	Average cell size (µm) from each percoll fraction ($n \geq 300$ cells)
60	1.12 ± 0.27
62	1.17 ± 0.32
64	1.29 ± 0.35
66	1.54 ± 0.39
68	Very few or no cells
70	Very few or no cells
72	Very few or no cells
74	Very few or no cells
76	Very few or no cells

The percoll step-gradient used for *Mtb* cells was 60-76%. The 68-76% percoll fractions of the *Mtb* sample contained very few or no cells. The 60%, 62% and 64% *Mtb* percoll fractions were also mostly composed of shorter cells based on their frequency of length distribution (**Figure 8C**). However, since the average size of cells from 60% and 62% were comparable, they were mixed together and termed *Mtb* SCF1. Subsequently, the 64% *Mtb* percoll fraction was termed as *Mtb* SCF2. The 66% percoll fraction was mostly composed of longer cells and was called *Mtb* NCF (**Figure 8C**).

Supplementary Table S7. The average size ranges (± SD) and proportions of *Mtb* cells from SCF1, SCF2, NCF, and the outliers in each fraction, as measured under bright-field (BF) and DIC, post acid-fast staining (n ≥ 300 cells)

Average cell length / Proportion Measurements of *Mtb* Cells [Length Measured Under Bright-field (BF) or DIC]			
Name and % of the Percoll Fraction	Length (µm) Average± SD	Proportion	Proportion of Outliers
SCF1 (60 & 62% Percoll)	1.14 ± 0.29 (BF)	68.55%	31.45%
	1.06 ± 0.24 (DIC)	72.49%	27.51%
SCF2 (64% Percoll)	1.29 ± 0.35 (BF)	69.69%	30.31%
	1.41 ± 0.37 (DIC)	70.97%	29.03%
NCF (66% Percoll)	1.54 ± 0.39 (BF)	67.09%	32.97%
	1.64 ± 0.47 (DIC)	71.54%	28.46%

The lines in the first column indicate the percoll fractions; the average lengths and SD of cells in the SCF1, SCF2, and NCF fractions as measured under bright-field (BF) (top lines) and under DIC (bottom lines) are indicated in the second column; their respective proportions are mentioned in the third column; the proportion of outliers which are much above or below the average length ± SD is indicated in the fourth column. These are cells, which were also counted to determine the average length, but having lengths much lower or much higher than the average length of the cells in the respective fraction, in spite of having same density as that of average sized cells. n ≥ 300 cells from each fraction.

SUPPLEMENTARY TEXT

CFU Determination of *Msm* and *Mtb* SCF1, SCF2 and NCF

The cells in the *Msm* 64% (SCF1), *Msm* 66% (SCF2) and *Msm* 78% (NCF), obtained following analytical scale percoll gradient centrifugation, were resuspended in 400 µl of 1x PBS or 0.5% Tween 80 or Middlebrook 7H9 medium, while the cells in the NCF were further diluted 250 times with Middlebrook 7H9 medium (as mentioned under 'MATERIALS AND METHODS'). Subsequently, 200 µl from each of the respective cell suspensions was added into 25 ml Middlebrook 7H9 medium taken in 100 ml flask, to obtain 10^3 cells/ml of cell density, followed by exposure to stress. In order to obtain 10^5 cells/ml of the preparative scale percoll gradient fractionated SCF and NCF [which was visually made (by dilution with the medium) to the same cell density as that of SCF (the 400 µl of SCF1 + SCF2 mixture prepared)], 100 µl from each of the respective cell suspensions was added into 25 ml Middlebrook 7H9 medium taken in 100 ml flask followed by exposure to the stress (as mentioned under 'MATERIALS AND METHODS'). It may be noted here that the visual comparison of cell density of the fractions for matching cfu were earlier verified for the accuracy of cfu values by plating performed multiple times using independent samples prepared from multiple cultures at different times by different people. Following the addition of cells from the cell suspensions, 100 µl was taken from each of these samples from the 25 ml Middlebrook 7H9 medium, at 0 hr (before exposure) and at the time mentioned post-exposure to the stress agents, for serial dilution followed by plating to determine their cfu.

Since the cells in the percoll fractions were found to elongate, when kept in PBS or Middlebrook 7H9 medium after removal of percoll, it was not possible to take cell count of the fractions or to determine cfu (takes 3 days), to obtain almost equal cell number of the SCF1, SCF2 and NCF for the exposure to stress agents. Therefore, a large number of sets (n = 80 sets) of the SCF1, SCF2, and NCF samples, which were prepared independently on multiple occasions, were plated to find out the consistency and reproducibility of the size range of the cells that get fractionated into 64%, 66%, and 78% percoll fractions, corresponding to SCF1, SCF2, and NCF samples, respectively. From these experiments, the consistent volumes of the SCF1, SCF2, and NCF, which reproducibly gave consistent cfu, were found out and used.

The average (± standard deviations) observed for the *Msm* cfu in the SCF1, SCF2 and NCF were as follows: 110.4 (± 43.3), 202.6 (± 80.03) and 124.6 (± 94.7). Though these variations were observed in the cfu, the technical triplicates within each set of experiment were consistent and the nature and the trend of the response of the individual fractions were reproducible and consistent.

The cell density of the *Mtb* SCF2 (64%) and *Mtb* NCF (66%) was visually made (by dilution with the medium) to the same cell density as that of *Mtb* SCF1 (60 + 62%). In order to obtain 10^4 cells/ml of *Mtb* SCF1, SCF2 and NCF, for exposure to stress, 100 µl each of the respective cell suspensions was added into 25 ml Middlebrook 7H9 medium taken in 100 ml flask followed by exposure to the stress.

The average (± standard deviations) observed for the *Mtb* cfu in the SCF1, SCF2 and NCF were as follows: 42.8 (± 7.6), 57.2 (± 8.5) and 45.4 (± 21.9). Even though these variations were observed in the cfu, as observed in *Msm* cell samples, the technical triplicates within each set of experiment were consistent and the nature and the trend of the response of the individual fractions were reproducible and consistent.

It was not possible to obtain 100% enrichment for either the SCs or the NCs of *Msm* or *Mtb* cells in any percoll fraction for the following probable reasons. The cell size heterogeneity of *Msm* mother cells varied from on an average of 4 µm (normal size) to 9 µm (longer size) in length. With the cells elongating prior to division, the length varied from 8 µm to 18 µm! Therefore, although difficult to determine, it is possible that the longer cells whose sizes are

higher than the average cell size (+) SD in the 64% *Msm* SCF1 might have come from the asymmetric division of longer mother cells. Similarly, the shorter cells whose sizes are lesser than the average cell size (-) SD in the 78% *Msm* NCF might have come from the asymmetric division of shorter mother cells. Similar explanations may as well be applicable to the enrichment of *Mtb* cells in the respective percoll fractions.

We speculate that the low proportions of normal-sized cells in the SCF and the short cells in the NCF could probably be due to the comparable buoyant density of these respective minor population of the cells to that of the larger proportion of the cells in the respective fraction. Nevertheless, majority of the shorter cells got fractionated into the percoll fractions of low buoyant density. Differential buoyant density of mycobacterial cells has been found in *M. tuberculosis* cells subjected to multiple stress conditions and has been suggested to be due to differential lipid (triglyceride) content (Deb et al., 2009). Thus, the heterogeneity in the population seemed to be not confined to cell size alone, but to density as well, indicating that the high levels of heterogeneity found in mycobacterial population (Deb et al., 2009; McCarthy, 1974; Khomenko, 1987; Anuchin et al., 2009; Ghosh et al., 2009; Ryan et al., 2010; Farnia et al., 2010; Markova et al., 2012; Aldridge et al., 2012) seem to be based on several parameters, operating through multiple mechanisms, under diverse growth and stress conditions.

Equal cell density (10^3, 10^4, or 10^5 cells/ml) of the respective *Msm* and *Mtb* SCF1, SCF2, and NCF cells were exposed individually to a range of concentrations of rifampicin and isoniazid (antibiotic stress) and H_2O_2 (oxidative stress) (Milano et al., 2001), for different durations. Likewise, the *Msm* SCF1, SCF2 and NCF cells with the same cell density (10^3 cells/ml) were also exposed to 7.5 mM $NaNO_2$ (pH 5) (nitrite stress) (Colangeli et al., 2009) for 30 min (as mentioned under 'MATERIALS AND METHODS'). The percentage survival of the different samples, in terms of cfu, against the four stress agents was determined by plating the respective stressed cells and the unstressed cells on stress-agent-free plates. Using these experimental rationale and strategy, we investigated whether SCF1, SCF2, and NCF cells showed differential survivability against these stress agents.

Rationale for the Range of Rifampicin, Isoniazid and H_2O_2 Concentrations Used

In order to find out the robustness of the stress response exhibited by *Msm* SCF1, SCF2 and NCF cells, the cells were exposed to a range of rifampicin, isoniazid and H_2O_2 concentrations. Subsequently, the response of the cells in these percoll fractions to acidified nitrite stress, was also determined. However, the concentrations of these stress conditions which resulted in very less lethality or those which effected in very high lethality to the cells, were not selected for the stress exposure. The range of concentrations of rifampicin, isoniazid and H_2O_2 used are those that effect survivability between 0% and 100% survival. For example, since the survivability of the *Msm* NCF cells was ~80% (data not shown) when exposed to < 25 µg/ml rifampicin and < 10% survival during 100 µg/ml rifampicin exposure, these extreme rifampicin concentrations were not used for the experiments. In the same manner, exposure of *Msm* NCF cells to < 2.5 µg/ml isoniazid concentration showed ~80% survival (data not shown). In contrast, the exposure of *Msm* SCF cells to 15 µg/ml isoniazid concentration resulted in < 10% survival. Likewise, the *Msm* NCF cells when exposed to 0.4 mM H_2O_2, the percentage survival was > 90%, while the exposure of the cells to 1 mM H_2O_2 resulted in < 10% survival. Hence, these extreme concentrations of rifampicin, isoniazid and H_2O_2 and were not used for the experiments. Similarly, exposure of *Msm* NCF cells to 10 mM $NaNO_2$ (pH 5) was observed to be lethal (data not shown). Hence, the *Msm* cell samples were exposed to 7.5 mM of acidified sodium nitrite for 30 min. The durations of exposure to the stress agents for the chosen range of concentrations were also standardised keeping the range of survival in view.

References

Aldridge, B. B., Fernandez-Suarez, M., Heller, D., Ambravaneswaran, V., Irimia, D., Toner, M., et al. (2012). Asymmetry and aging of mycobacterial cells lead to variable growth and antibiotic susceptibility. *Science* 335, 100-104.

Anuchin, A. M., Mulyukin, A. L., Suzina, N. E., Duda, V. I., EI-Registan, G. I., and Kaprelyants, A. S. (2009). Dormant forms of *Mycobacterium smegmatis* with distinct morphology. *Microbiology* 155, 1071-1079.

Colangeli, R., Haq, A., Arcus, V. L., Summers, E., Magliozzo, R. S., McBride, A., et al. (2009). The multifunctional histone- like protein Lsr2 protects mycobacteria against reactive oxygen intermediates. *Proc. Natl. Acad. Sci. U.S.A.* 106, 4414-4418.

Deb, C., Lee, C. M., Dubey, V. S., Daniel, J., Abomoelak, B., Sirakova, T. D., et al. (2009). A novel in vitro multiple-stress dormancy model for *Mycobacterium tuberculosis* generates a lipid-loaded, drug-tolerant, dormant pathogen. *PLoS ONE* 4, e6077.

Farnia, P., Mohammad, R. M., Merza, M. A., Tabarsi, P., Zhavnerko, G. K., Ibrahim, T. A., et al. (2010). Growth and cell-division in extensive (XDR) and extremely drug resistant (XXDR) tuberculosis strains: transmission and atomic force observation. *Int. J. Clin. Exp. Med.* 3, 308-314.

Ghosh, J., Larsson, P., Singh, B., Pettersson, B. M., Islam, N. M., Sarkar, S. N., et al. (2009). Sporulation in mycobacteria. *Proc. Natl. Acad. Sci. U.S.A.* 106, 10781-10786.

Khomenko, A. G. (1987). The variability of *Mycobacterium tuberculosis* in patients with cavitary pulmonary tuberculosis in the course of chemotherapy. *Tubercle* 68, 243-253.

Markova, N., Slavchev, G., and Michailova, L. (2012). Unique biological properties of *Mycobacterium tuberculosis* L-form variants: impact for survival under stress. *Int. Microbiol.* 15, 61-68.

McCarthy, C. (1974). Effect of palmitic acid utilisation on cell division in *Mycobacterium avium*. *Infect. Immun.* 9, 363-372.

Milano, A., Forti, F., Sala, C., Riccardi, G., and Ghisotti, D. (2001). Transcriptional regulation of *furA* and *katG* upon oxidative stress in *Mycobacterium smegmatis*. *J. Bacteriol.* 183, 6801-6806.

Ryan, G. J., Hoff, D. R., Driver, E. R., Voskuil, M. I., Gonzalez-Juarrero, M., Basaraba, R. J., et al. (2010). Multiple *M. tuberculosis* phenotypes in mouse and guinea pig lung tissue revealed by a dual-staining approach. *PLoS ONE* 5, e11108.

Snapper, S. B., Melton, R. E., Mustafa, S., Kieser, T., and Jacobs, W. R., Jr. (1990). Isolation and characterisation of efficient plasmid transformation mutants of *Mycobacterium smegmatis*. *Mol. Microbiol.* 4, 1911-1919.

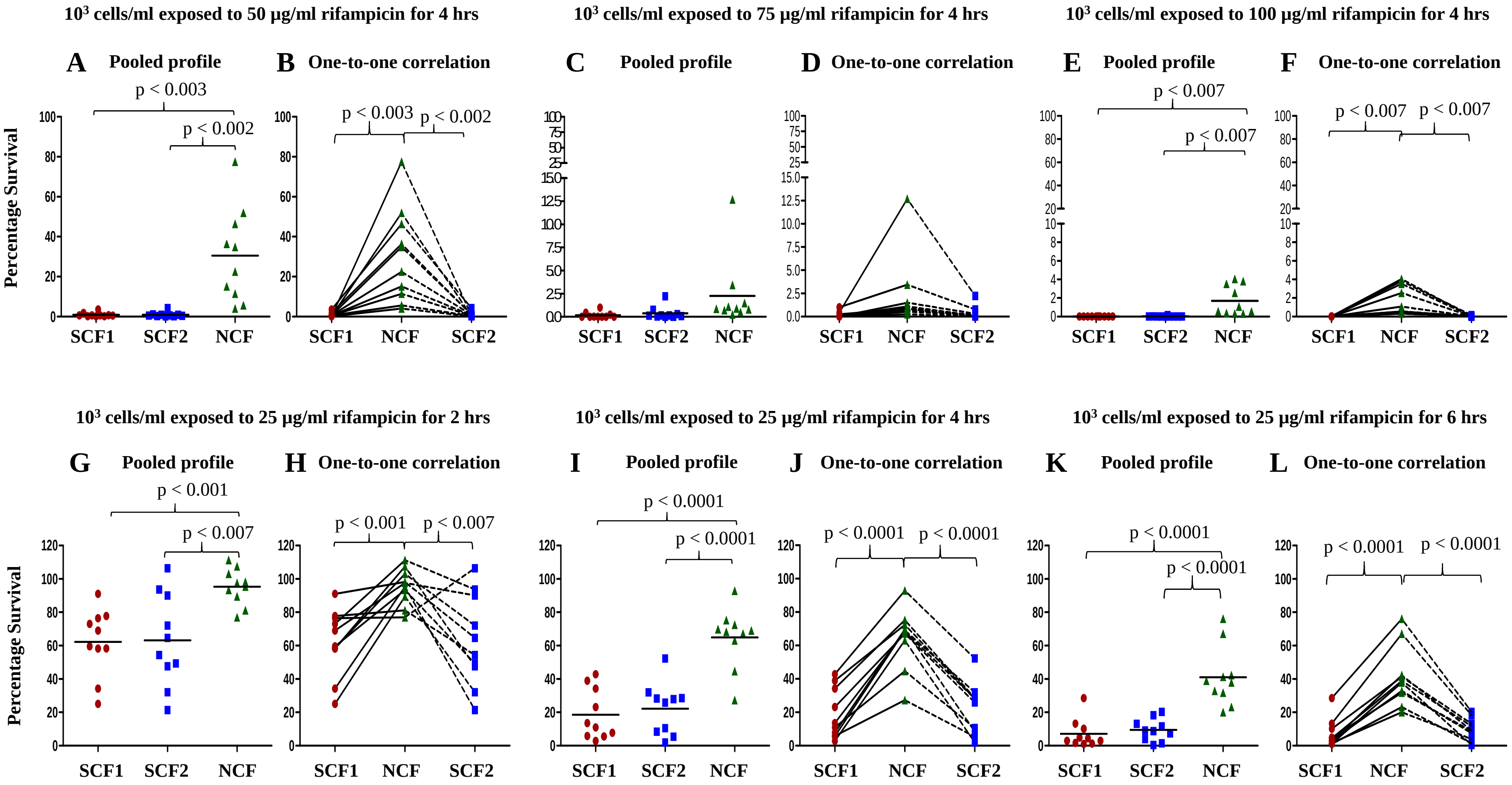

Supplementary Figure S1. Mean percentage survival of *Msm* SCF1, SCF2, and NCF cells when exposed to different concentrations of rifampicin and for different time durations. Percentage survival of 10^3 cells/ml of SCF1 and SCF2 in comparison to NCF when exposed to: **(A,B)** 50 µg/ml rifampicin; **(C,D)** 75 µg/ml rifampicin and **(E,F)** 100 µg/ml rifampicin for 4 hrs (n = 10 samples each). Percentage survival of SCF1 and SCF2 as compared to NCF when exposed to 25 µg/ml rifampicin for: **(G,H)** 2 hrs; **(I,J)** 4 hrs and **(K,L)** 6 hrs (n = 10 samples each). **(A,C,E,G,I,K)** Pooled results of all the experimental sets; **(B,D,F,H,J,L)** Relative one-to-one correlation of the survival of the samples in each independent set.

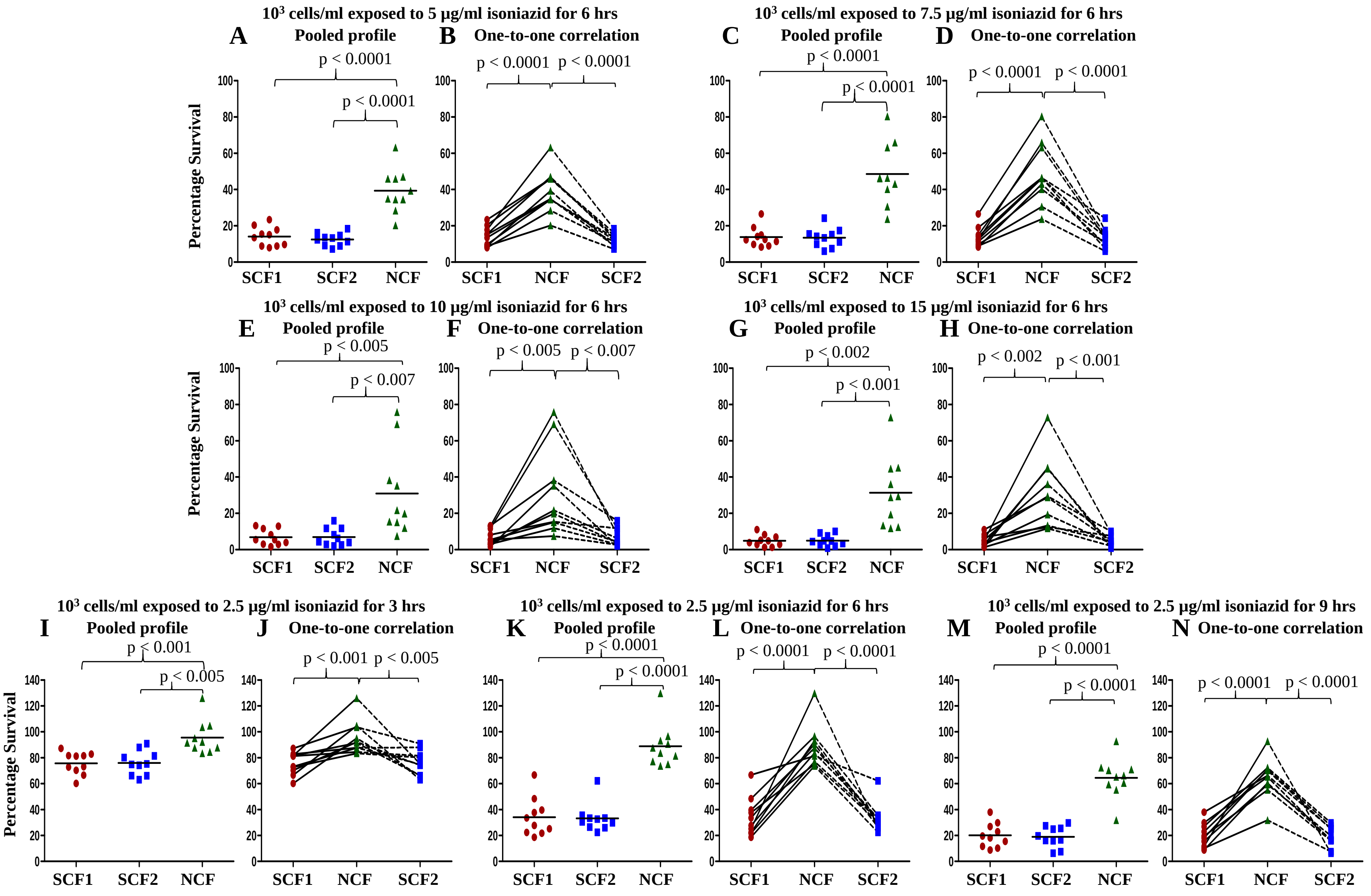

Supplementary Figure S2. Mean percentage survival of *Msm* SCF1, SCF2, and NCF cells when exposed to different concentrations of isoniazid and for different time durations. Percentage survival of 10^3 cells/ml of SCF1 and SCF2 in comparison to NCF when exposed to: **(A,B)** 5 µg/ml isoniazid; **(C,D)** 7.5 µg/ml isoniazid; **(E,F)** 10 µg/ml isoniazid and **(G,H)** 15 µg/ml isoniazid for 6 hrs (n = 10 samples each). Percentage survival of SCF1 and SCF2 as compared to NCF when exposed to 2.5 µg/ml isoniazid for: **(I,J)** 3 hrs; **(K,L)** 6 hrs and **(M,N)** 9 hrs (n = 10 samples each). **(A,C,E,G,I,K,M)** Pooled results of all the experimental sets; **(B,D,F,H,J,L,N)** Relative one-to-one correlation of the survival of the samples in each independent set.

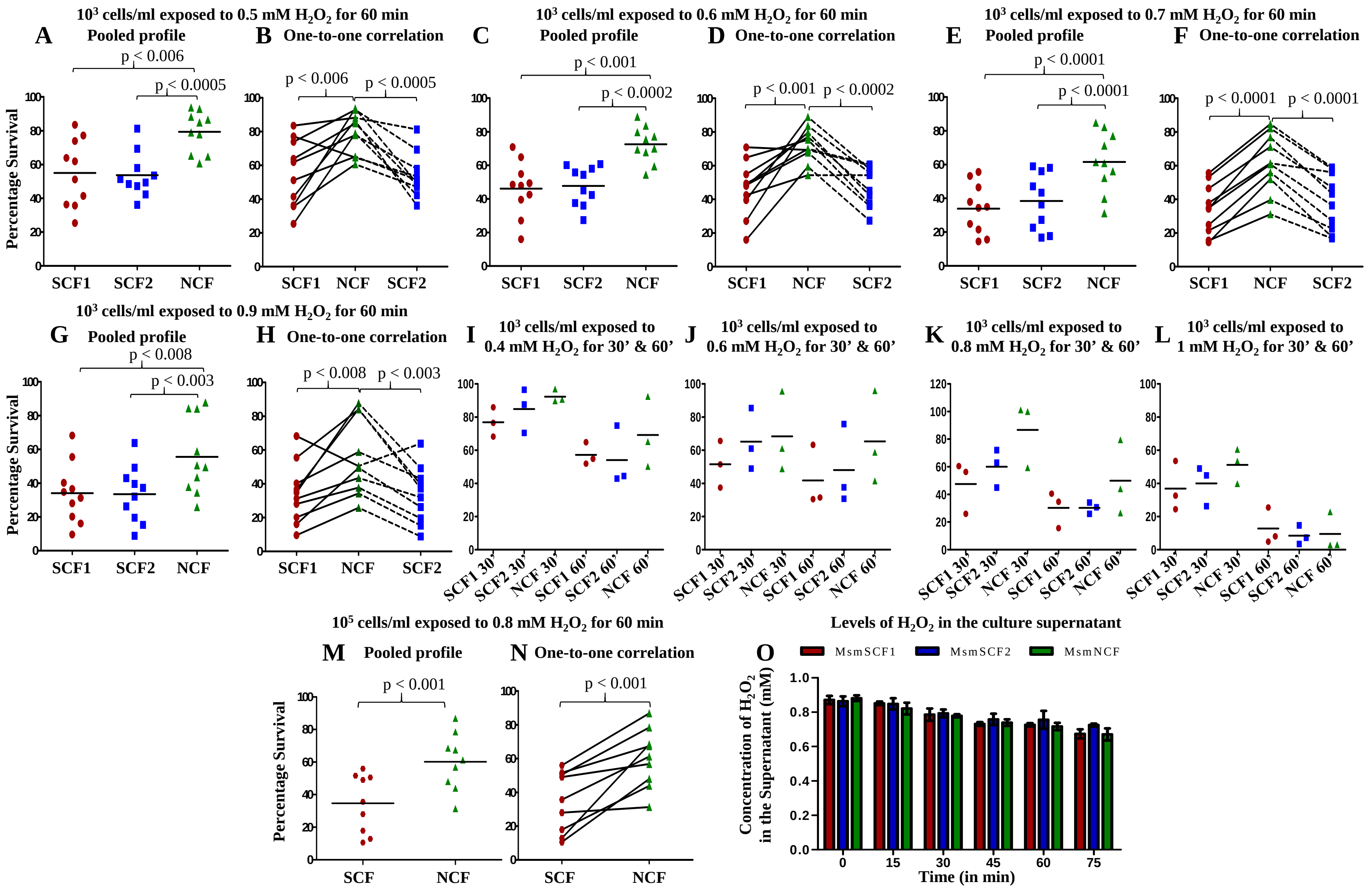

Supplementary Figure S3. Percentage survival of different densities of *Msm* SCF1, SCF2, and NCF cells, when plated after exposure to different concentrations of H_2O_2 for different time periods. (A-H) Percentage survival of 10^3 cells/ml of the different percoll fractions against different concentrations of H_2O_2 (0.5-0.9 mM H_2O_2, n = 10 samples each) for 60 min. **(I-L)** Percentage survival of 10^3 cells/ml of the different percoll fractions during the 30 min and 60 min incubations (n = 3 samples each) against a range of H_2O_2 concentrations. **(M,N)** Exposure of higher density (10^5 cells/ml) of the cells to 0.8 mM H_2O_2 for 60 min (n = 9 samples each). **(A,C,E,G,M)** Pooled results of all the experimental sets; **(B,D,F,H,N)** Relative one-to-one correlation of the survival of the samples in each independent set. **(O)** The concentration of H_2O_2 during the 75 min incubation of the cells in the presence of 0.8 mM H_2O_2.

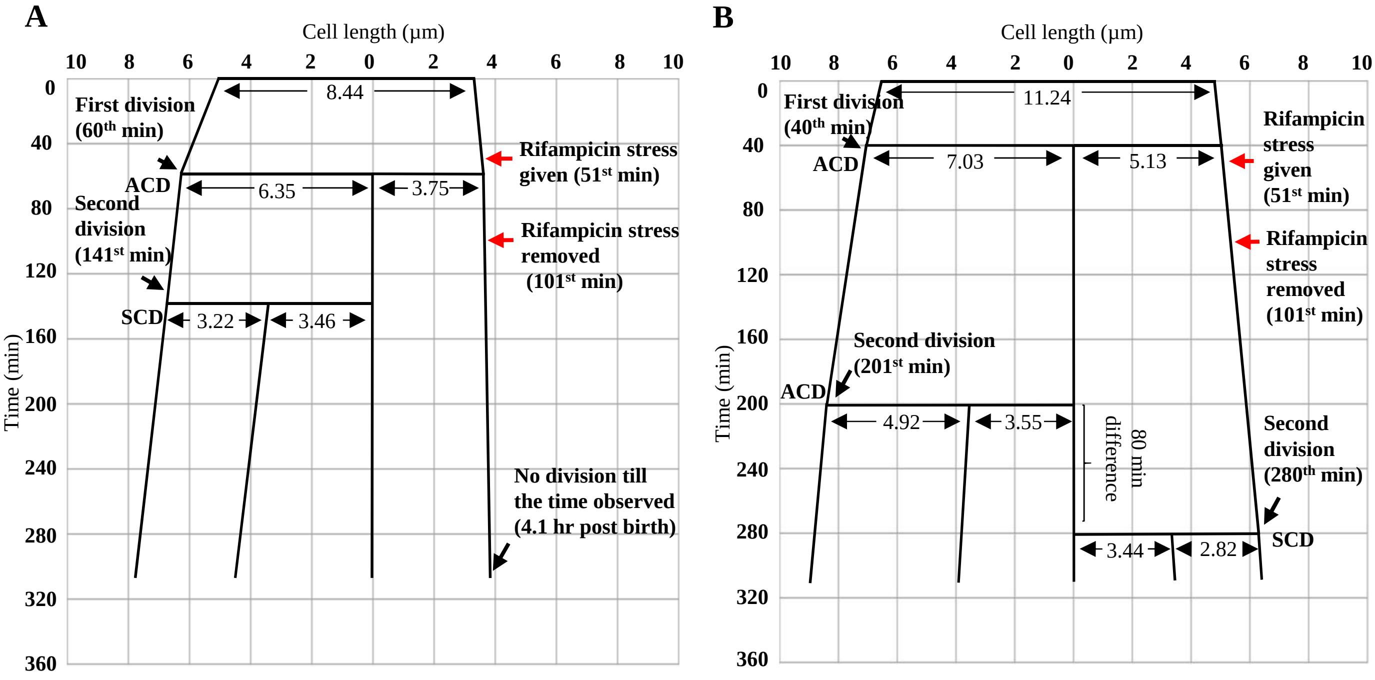

Supplementary Figure S4. **The lineage of the growth and division of rifampicin-stressed *Msm* normal/long-sized cells undergoing asymmetric division tracked during live cell-time lapse imaging.** The zero time point does not correlate with the birth of the starting mother cell. The cell lengths given are from the DIC images, but not drawn to scale. The time of generation of daughter cells from asymmetric cell division (ACD) and symmetric cell division (SCD) has been indicated for each division. The time point of exposure of stress: **(A,B)** 12.5 µg/ml rifampicin for 50 min and its withdrawal have been indicated with red arrows.

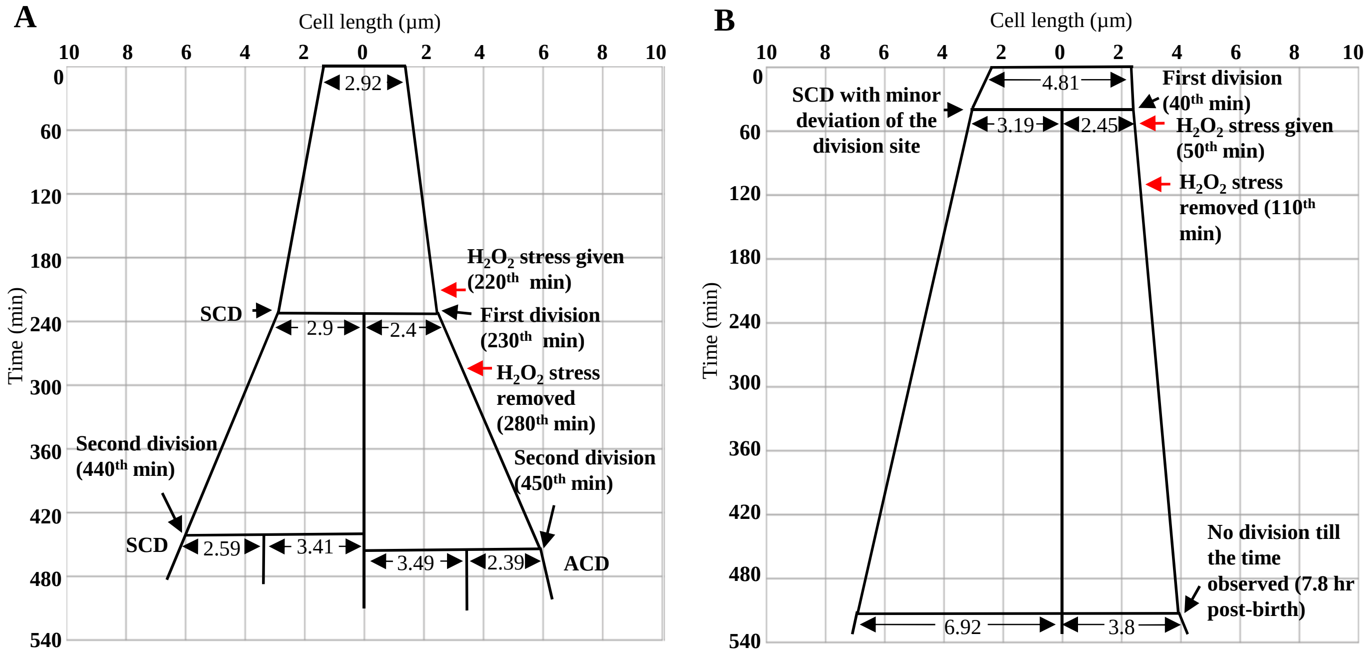

Supplementary Figure S5. **The lineage of the growth and division of H_2O_2-stressed *Msm* short cells undergoing symmetric division tracked during live cell-time lapse imaging.** The zero time point does not correlate with the birth of the starting mother cell. The cell lengths given are from the DIC images, but not drawn to scale. The time of generation of daughter cells from asymmetric cell division (ACD) and symmetric cell division (SCD) has been indicated for each division. The time point of exposure of stress: **(A,B)** 0.8 mM H_2O_2 for 1 hr and its withdrawal have been indicated with red arrows.

Supplementary Video Legends

Video S1. Time lapse microscopy of symmetric and asymmetric divisions of *Msm* short cells. Live cell time-lapse microscopy of an *Msm* short cell undergoing successive symmetric and highly-deviated ACDs. First, the short cell (3.59 µm) undergoes symmetric division to generate two equal-sized daughter cells of lengths, 2.40 µm and 2.04 µm. The shorter daughter cell further went for highly-deviated ACD to generate two unequal-sized daughter cells of lengths, 2.70 µm and 1.70 µm, respectively. The longer daughter cell underwent symmetric division to generate two equal-sized daughter cells of lengths, 2.11 µm and 2.17 µm, respectively. The images were captured under DIC at 10 min interval for 12 hrs.

Video S2. Time lapse microscopy of the susceptibility of sister daughter cells from asymmetrically dividing mycobacteria to rifampicin. Representative time lapse microscopy of *Msm* growing on agarose pad with Middlebrook 7H9 medium. 12.5 µg/ml rifampicin exposure was given for 50 min in agarose pad, t = 3:10-4:00 (red square), replaced with Middlebrook 7H9 medium after t = 4:00 and monitored for 10 hrs. Cell, marked with blue color, underwent asymmetric division generating daughter cells of length 4.16 µm (pink) and 3.02 µm (yellow) at t = 3:50.

Video S3. Time lapse microscopy of the susceptibility of sister daughter cells from symmetrically and asymmetrically dividing mycobacteria when exposed to 0.8 mM H_2O_2. Representative time lapse microscopy of *Msm* growing on agarose pad with Middlebrook 7H9 medium. H_2O_2 exposure was given for 1 hr in agarose pad, t = 00:50-1:50 (red square), replaced with Middlebrook 7H9 medium after t = 1:50 and monitored for 8 hrs. Cell at the left side, [pink (t = 0)] underwent symmetric division, generating two equal sized daughter cells 3.46 µm (maroon) and 3.48 µm (cyan) at t = 1:00. Cell at the right side, [green (t = 0)] underwent asymmetric division generating normal-sized cell 4.81 µm (red) and short cell 3.1 µm (pink) at t = 00:30.

Video S4. Time lapse microscopy of the susceptibility of sister daughter cells from asymmetrically dividing mycobacteria to 0.8 mM H_2O_2. Representative time lapse microscopy of *Msm* growing on agarose pad with Middlebrook 7H9 medium. H_2O_2 exposure was given for 1 hr in agarose pad, t = 00:50-1:50 (red square), replaced with Middlebrook 7H9 medium after t = 1:50 and monitored for 8 hrs. Cell, marked with blue color, underwent asymmetric division generating normal-sized cell 4.35 µm (green) and short cell 2.47 µm (pink) at t = 1:30.

Video S5. Time lapse microscopy of the susceptibility of sister daughter cells from asymmetrically and symmetrically dividing mycobacteria to 0.8 mM H_2O_2. Representative time lapse microscopy of *Msm* growing on agarose pad with Middlebrook 7H9 medium. H_2O_2 exposure was given for 1 hr in agarose pad, t = 1:09-2:10 (red square), replaced with Middlebrook 7H9 medium after t = 2:10 and monitored for 6 hrs. Cell at the left side, [blue (t = 0)] underwent asymmetric division, generating normal sized cell 4.01 µm (green) and short cell 2.25 µm (red) at t = 00:59. Cell at the right side, [pink (t = 0)] underwent symmetric division, generating two daughter cells of length 4.61 µm (green) and 3.49 µm (blue) at t = 1:30.

Video S6. Time lapse microscopy of the susceptibility of sister daughter cells from symmetrically dividing mycobacteria to 0.8 mM H_2O_2. Representative time lapse

microscopy of *Msm* growing on agarose pad with Middlebrook 7H9 medium. H_2O_2 exposure was given for 1 hr in agarose pad, t = 1:09-2:10 (red square), replaced with Middlebrook 7H9 medium after t = 2:10 and monitored for 6 hrs. Cell, marked with blue color, underwent symmetric division generating daughter cells of length 3.85 µm (green) and 3.21 µm (pink) at t = 1:30.
